# Enabling QALY estimation in mental health trials and care settings: mapping from the PHQ-9 and GAD-7 to the ReQoL-UI or EQ-5D-5L using mixture models

**DOI:** 10.1007/s11136-023-03443-9

**Published:** 2023-06-14

**Authors:** Matthew Franklin, Monica Hernández Alava

**Affiliations:** Health Economics and Decision Science (HEDS), School of Health and Related Research (ScHARR), University of Sheffield, Regent Court, 30 Regent Street, Sheffield, S1 4DA UK

**Keywords:** Mapping, QALY, EQ-5D-5L, ReQoL-UI, GAD-7, PHQ-9, Mental health, Anxiety, Depression, Economic evaluation

## Abstract

**Purpose:**

Patient-reported outcome measures (PROMs) are commonly collected in trials and some care settings, but preference-based PROMs required for economic evaluation are often missing. For these situations, mapping models are needed to predict preference-based (aka utility) scores. Our objective is to develop a series of mapping models to predict preference-based scores from two mental health PROMs: Patient Health Questionnaire-9 (PHQ-9; depression) and Generalised Anxiety Questionnaire-7 (GAD-7; anxiety). We focus on preference-based scores for the more physical-health-focussed EQ-5D (five-level England and US value set, and three-level UK cross-walk) and more mental-health-focussed Recovering Quality-of-Life Utility Index (ReQoL-UI).

**Methods:**

We used trial data from the Improving Access to Psychological Therapies (IAPT) mental health services (now called NHS Talking Therapies), England, with a focus on people with depression and/or anxiety caseness. We estimated adjusted limited dependent variable or beta mixture models (ALDVMMs or Betamix, respectively) using GAD-7, PHQ-9, age, and sex as covariates. We followed ISPOR mapping guidance, including assessing model fit using statistical and graphical techniques.

**Results:**

Over six data collection time-points between baseline and 12-months, 1340 observed values (*N* ≤ 353) were available for analysis. The best fitting ALDVMMs had 4-components with covariates of PHQ-9, GAD-7, sex, and age; age was not a probability variable for the final ReQoL-UI mapping model. Betamix had practical benefits over ALDVMMs only when mapping to the US value set.

**Conclusion:**

Our mapping functions can predict EQ-5D-5L or ReQoL-UI related utility scores for QALY estimation as a function of variables routinely collected within mental health services or trials, such as the PHQ-9 and/or GAD-7.

**Supplementary Information:**

The online version contains supplementary material available at 10.1007/s11136-023-03443-9.

## Introduction

Quality-adjusted life years (QALYs) are a popular metric to evaluate the cost-effectiveness of care interventions [1–4]. However, a common evidence gap exists between available clinical measures of effect and the detailed preference-based information (e.g. utility scores) needed to estimate QALYs [5]. Within mental health trials, patient-reported outcome measures (PROMs) like the Patient-Health Questionnaire-9 (PHQ-9) and Generalised Anxiety Disorder-7 (GAD-7) are commonly used (often together) to capture depression and anxiety severity, respectively [6–8]. These measures are also routinely collected by mental health services such as Improving Access to Psychological Therapies (IAPT) services (now called NHS Talking Therapies) in England as part of their patient-based performance metrics [6, 8–10]. However, such PROMs do not have preference-based value sets to enable cost-per-QALY estimates to be interpreted relative to thresholds to infer cost-effectiveness, e.g. in England and Wales, the National Institute for Health and Care Excellence’s (NICE’s) £20,000 to £30,000 per QALY threshold [4, 11, 12].

Preference-based PROMs like the EQ-5D three-level (EQ-5D-3L) and five-level (EQ-5D-5L) versions have country-specific preference-based value sets for the estimation of QALYs and are favoured by health technology assessment organisations internationally, including NICE [1–4]. However, existing empirical evidence indicates limitations of the EQ-5D measures in mental health populations, recommending a more mental health focussed preference-based measure for mental health service users [13–20]. The Recovering Quality-of-Life 20-item (ReQoL-20) and 10-item (ReQoL-10) are two such PROMs capturing ‘recovery-focussed quality-of-life’ for mental health service users [21]. A UK preference-based value set has been developed to calculate QALYs from seven ReQoL-10 items: the ReQoL Utility Index (ReQoL-UI) [22]. Key differences in ReQoL-UI and EQ-5D-5L design, utility score distributions, psychometric properties, and subsequently estimated QALYs have been assessed and discussed [23, 24].

Preference-based measures like the EQ-5D-5L or ReQoL-UI are frequently absent from clinical studies or routine service data collection, which prevents direct QALY calculation. The term ‘mapping’ is used to describe the process of estimating a statistical relationship between observed clinical outcome measures and preference-based measures using an estimation dataset containing both types of information. The estimated ‘mapping’ model can predict missing preference-based scores for clinical studies or care services based on observed clinical outcome measures. However, the distribution of preference-based scores tend to exhibit characteristics that make standard regression-based models such as linear and Tobit regressions inappropriate for mapping and their use should be discouraged, despite traditionally being common practice [25–27]. Specifically for mapping, adjusted limited dependent variable mixture models (ALDVMMs) were first proposed by Hernández Alava et al. [28] to deal with the distributional features presented by the EQ-5D-3L, with supportive evidence when modelling other preference-based scores such as EQ-5D-5L [26, 29]. Alternative mixture models, such as mixture beta regression models (Betamix), might also have benefits relative to ALDVMMs dependent on the utility scores underlying distribution [30–32].

Our overall aim is to map from the GAD-7 and PHQ-9 to the ReQoL-UI or EQ-5D-5L based on ‘best practice’ mapping methods using an estimation dataset obtained from an IAPT-based trial population [24, 33, 34]. To accomplish this aim, we firstly use ALDVMMs to map from the GAD-7 and PHQ-9 to the ReQoL-UI to enable QALY estimation. Secondly, the availability of the EQ-5D-5L in the estimation dataset provides an opportunity to investigate previously raised issues around the appropriateness of mapping from PHQ-9 and GAD-7 to generic measures such as the EQ-5D-5L [16]. This second objective is complicated by the fact EQ-5D-5L responses can be assigned utility scores using country-specific value sets, such as the current EQ-5D-5L value set for England (VSE) or United States value set (USVS), or predicted EQ-5D-3L utility scores using an existing mapping function [35–37]. In England and Wales, NICE does not recommend the VSE, instead previously recommending the ‘cross-walk’ by van Hout et al. [36]; however, since January 2022, NICE changed its recommendation from the cross-walk to the mapping function developed by the NICE Decision Support Unit (DSU) [4, 38–40]. Work is ongoing to recommend the most appropriate way to map to the DSU mapping function, and is therefore not included in our analysis. Instead, mapping to three EQ-5D-5L utility scores (i.e. VSE, USVS, and cross-walked) provide additional insights into the suitability of mapping to generic preference-based measures given the marked differences across their distributions [23, 41–43].

## Outcome measures

Appendix S1 provides a summarised overview of all PROMs.

### Mental health measures

The PHQ-9 is a self-reported screening for depression measure reflecting the Diagnostic and Statistical Manual of Mental Disorders, Fourth Edition—Text Revision (DSM–IV–TR) criteria [8, 44, 45]; summary score range: 0 (minimal depression) to 27 (severe depression).

The GAD-7 is a self-reported symptoms and severity of anxiety measure based on the DSM-IV GAD diagnostic criteria [7]; summary score range: 0 (minimal anxiety) to 21 (severe anxiety).

The PHQ-9 and GAD-7 are commonly used together to measure depression and anxiety symptomology, given the often comorbid nature of depression and anxiety [46, 47]. For example, IAPT services have operationalised the aforementioned based on ‘caseness’ (PHQ-9 ≥ 10; GAD-7 ≥ 8) and ‘reliable improvement’ (PHQ-9 absolute change ≥ 6; GAD-7 absolute change ≥ 4) threshold values as part of IAPT’s patient-based performance outcomes [6, 8–10]. As such, the measures’ summary scores (but not always the item scores) are routinely recorded for IAPT patients.

### Target measures and utility scores

#### ReQoL-UI

The ReQoL-UI classification system is based on seven ReQoL-10 items each with five severity levels, covering seven themes of self-reported recovery-focused quality-of-life [22]: autonomy; well-being; hope; activity; belonging and relationships; self-perception; physical health. The ReQoL-UI is described as having two overall dimensions: a mental health (six items) and a physical health (one item) dimension [22]. The ReQoL-UI represents (7^5^) 78,125 possible health states, with a score range from − 0.195 (worst state) to 1 (best state).

#### EQ-5D-5L

The EQ-5D-5L is a self-reported, generic health measure with five severity levels, over five dimensions/items: mobility; self-care; usual activity; pain/discomfort; anxiety/depression [48, 49]. The EQ-5D-3L is a previous version of the instrument which uses the same dimensions but with only three severity levels. The EQ-5D-5L’s classification system is able to represent (5^5^) 3,125 health states, compared to the EQ-5D-3L’s (3^5^) 243 health states. EQ-5D-5L utility scores can be estimated using either a direct value set or through using a mapping (‘cross-walk’) function to a EQ-5D-3L value set [35, 36]. Here we focus on two value sets, VSE and USVS, and the van Hout et al. [36] ‘crosswalk’ which maps to the EQ-5D-3L UK value set.

The cross-walk used a non-parametric response mapping method to predict values that are linked to the EQ-5D-3L value set. This method is based on independent cross-tabulations of EQ-5D-3L and EQ-5D-5L for each dimension and some assumptions about the allowable response patterns. In particular, it is assumed that any response at the lowest (highest) severity level of EQ-5D-5L always corresponds to a response at the lowest (highest) severity level of EQ-5D-3L; therefore, the cross-walk produces a EQ-5D-5L value set with the same range as the EQ-5D-3L UK value set, ranging from 1 (best state) to − 0.594 (worst state). As such, cross-walked utility scores mildly mimic distributional aspects of the original EQ-5D-3L UK value set [50].

In comparison, the VSE’s and USVS’s value range is smaller than the EQ-5D-3L’s/cross-walk’s, from − 0.285 or − 0.573 (worst state) to 1 (best state), respectively, when assigned to the EQ-5D-5L’s 3125 health states.

## Methods

### Pre-mapping considerations: conceptual overlap and existing mapping studies

An important pre-mapping consideration suggested by ISPOR guidance is the extent of overlap between the clinical outcomes measures and target preference-based measure/score; if there is little overlap, mapping success is unlikely [34]. Measures’ conceptual and practical overlap can be examined using psychometric methods (for example assessing correlations and effects sizes) and additional learnings derived from previous mapping studies.

In terms of psychometrics, EQ-5D measures’ results offer better support in common mental health disorders such as anxiety and depression compared to severe disorders like schizophrenia and bipolar disorder [16–19, 51]. Relatedly, the ReQoL-UI’s and EQ-5D-5L’s relative psychometric properties have been assessed in general and mental health populations [24, 52]. Against the PHQ-9 and GAD-7 in IAPT patients, Franklin et al. [24] concluded the ReQoL-UI has relatively better construct validity with the PHQ-9; however, the EQ-5D-5L had relatively better construct validity with the GAD-7.

The mapping literature is sparse in this area, limiting the insights that can be obtained. A 2019 systematic review of mapping studies by Mukuria et al. [25] identified a single study focussed on mapping from mental health measures (e.g. PHQ-9 and GAD-7) to preference-based measures (EQ-5D-3L and SF-6D) [16]: Brazier et al. [16] questioned the appropriateness of mapping from mental health measures to generic preference-based measures based on their mapping performance statistics. However, Brazier et al. [16] analyses did not include mixture models, rather they focussed on more traditional OLS, Tobit, and response-level mapping models. One other study ‘mapped’ from the PHQ-9 to the EQ-5D-3L using a non-regression-based approach (i.e. equipercentile linking), however, limited reported results restricted performance assessment of this approach [53–55]. A non-peer-reviewed study mapped from the Health of Nation Outcomes Scale (HoNOS) to the ReQoL-UI, which is the only previous study we identified which mapped to the ReQoL-UI; however, this study only used an OLS model and the HoNOS is clinician not patient-reported, which may have contributed to the authors suggesting caution when using their mapping functions.

### Estimation data source

The estimation dataset was obtained from a parallel-groups, randomised waitlist-controlled trial examining the effectiveness and cost-effectiveness of internet-delivered Cognitive Behavioural Therapy (iCBT) for patients presenting with depression and anxiety, conducted at an established IAPT service with eligibility criteria described in Appendix S1 [33, 56]. The trial collected PROM data at baseline and 8-week across both trial-arms; additional data collection time-points for the intervention-arm only were at 3-, 6-, 9-, and 12-months. NHS England Research Ethics Committee provided trial ethics approval (REC Reference: 17/NW/0311). The trial was prospectively registered: Current Controlled Trials ISRCTN91967124. The trial is completed with the protocol and main results published [23, 33, 56].

### Mapping models

Our mapping of interest is fitting ALDVMMs to the ReQoL-UI and EQ-5D-5L (VSE, USVS, or cross-walk); all utility scores are UK/England specific, apart from the USVS. When the predictions from ALDVMMs were deemed to not sufficiently suit the observed data, Betamix models were used instead. We used the *aldvmm* or *betamix* command within the statistical software package Stata Version 17 [57]. The *aldvmm* command estimates the variant of the model presented in Hernández Alava et al. [27, 58]. Full instructions on how to use the *aldvmm* command are described by Hernández Alava and Wailoo [29]. The *betamix* command is described by Gray and Hernández Alava [31].

ALDVMMs are flexible models that can approximate many distributional forms by combining (mixing) multiple component distributions; each component’s distribution is allowed to have different parameters for the same set of variables (i.e. xvars). Additional probability variables (i.e. pvars) predict the probability of each observation belonging to each component. Betamix models are similar to ALDVMMs in terms of being mixture models; although, key differences are that they are designed for dependent variables bounded in an interval (i.e. beta distributions are bounded between 0 and 1) and there are additional modelling options such as being able to specify a probability mass (i.e. pmass) at the lower and upper score, and some defined truncation point, of the dependent variable.

We estimated ALDVMMs (and Betamix when required) with 2–4 components; although it is possible to estimate 1-component models, fitting more than 1-component tends to improve model fit so we don’t present the 1-component model results. We describe how we moved from 2 to 4 component models in Appendix S1. For all ALDVMMs, we included PHQ-9 summary score (continuous variable), GAD-7 summary score (continuous variable), age (continuous variable), and sex (binary variable) to predict the utility scores within the components; however, we evaluate models with different variables and specifications. When a Betamix was chosen as preferable, only the PHQ-9 and GAD-7 summary scores were included as the core covariates of interest given the additional computational time and complications of trying to assess more modelling specifications using Betamix relative to ALDVMMs.

### Model fit statistics and graphs

To compare results across models, we considered standard model fit measures/criteria such as absolute mean error (AE), mean absolute error (MAE), root mean square error (RMSE), log likelihood (LL), Akaike information criteria (AIC), Bayesian information criteria (BIC), and graphical methods for model selection in mapping [59]. An AE closer to zero, higher LL, and lower MAE, RMSE, AIC, and BIC indicated a better fit. Graphical methods have been shown to be essential for mapping model selection as described in Appendix S1 [59]; due to the number of models included in this mapping study which produced a large number of graphs, we only compare graphs between two models based on any given target utility score after assessing their model fit statistics. Specifically, we plotted the mean of the predicted utility scores with the mean observed values by PHQ-9 and GAD-7 scores. We also simulated data from the models and plotted the cumulative distribution functions (CDFs) comparing simulated with observed data across the severity range.

Throughout we followed ISPOR good practice mapping guidance [34]. As ISPOR good practice mapping guidance does not wholly support the use of internal validation approaches (i.e. splitting the dataset into an estimation and validation dataset), in part because sample splitting means a reduced sample size for estimation and there is uncertainty around what extra value the information these validation analyses provide, we have opted to not split the dataset for such an internal validation approach [34].

## Results

### Descriptive statistics of the estimation dataset population

Overall, 353 people at baseline across both trial-arms (237 intervention; 116 control) completed the ReQoL-10, GAD-7, and PHQ-9; 352 completed the EQ-5D-5L. Across all six data collection time-points, 1340 observed value scores for each of the ReQoL-UI, GAD-7, and PHQ-9 were available; 1339 for the EQ-5D-5L. All observed case data across all time-points and trial-arms were used for mapping.

**Fig. 1:**
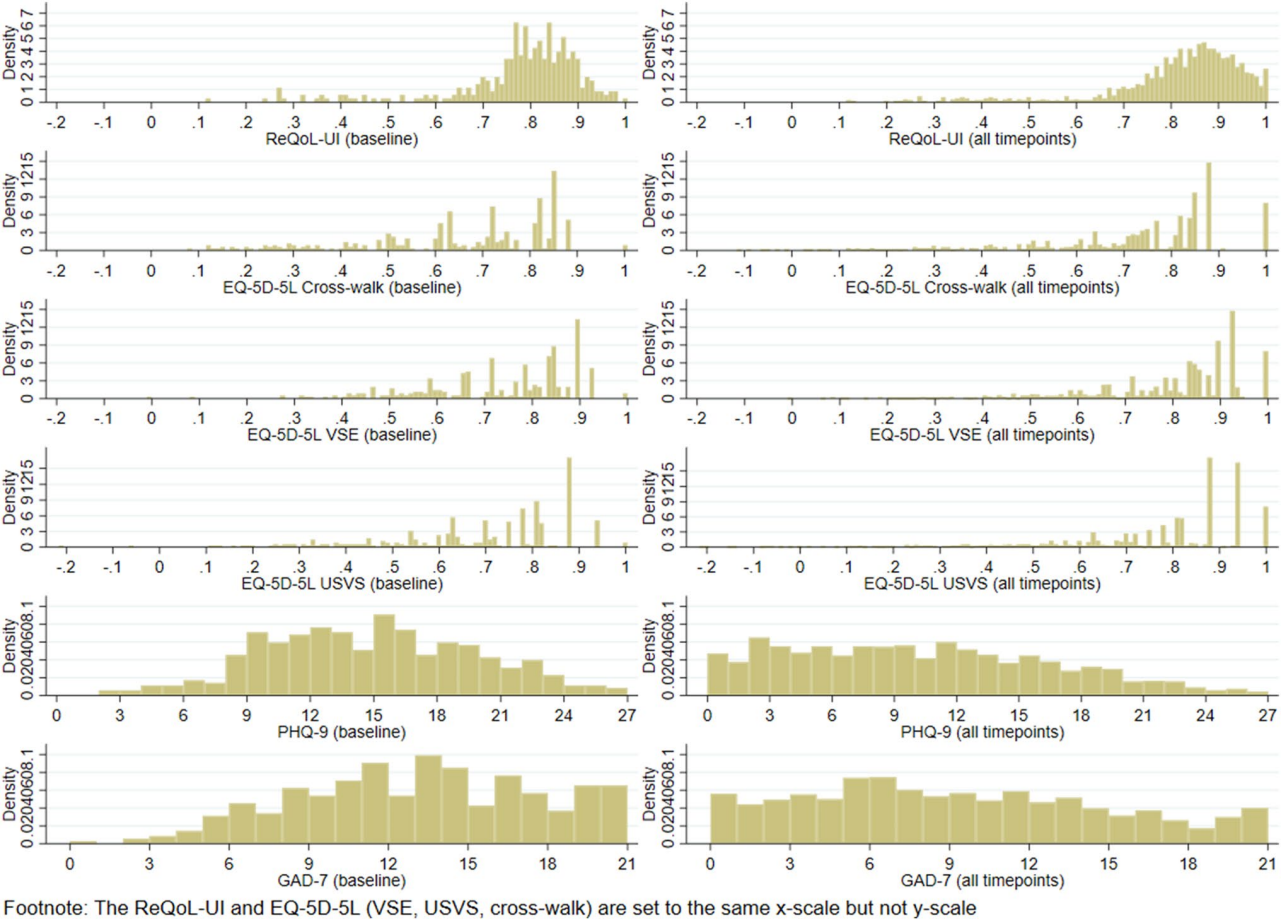
Distribution of ReQoL-UI, EQ-5D-5L VSE, USVS and cross-walk, PHQ-9, and GAD-7 scores at baseline and across all time-points

The sample mean age at baseline is 33 (range: 18–74) with a female majority (71%). Figure 1 presents the distributions of PROM scores, with comparisons of ‘baseline’ vs ‘all time points’ distributions showing a sample shift towards the healthier part of the distributions. The ReQoL-UI has a smoother distribution than EQ-5D-5L utility scores. Additional descriptive statistics are provided in Appendix S1.

### Model fit statistics

Model fit statistics for 36 ALDVMMs models are presented in Table 1: 12 ALDVMMs to each of the ReQoL-UI, EQ-5D-5L VSE and cross-walk. Generally, across all models, increasing the number of components improved model fit and there were no perceived issues with the use of ALDVMMs.

**Table 1:** Model fit statistics for the ALDVMMs for the ReQoL-UI, EQ-5D-5L VSE and cross-walk

No	Target	P-var	Obs	LC	DF	Mean	Min	Max	LL	AIC	BIC	AE	MAE	RMSE
R1	ReQoL-UI	PHQ-9, GAD-7, age, sex	1340	2	17	0.8182	0.5401	0.9489	1468.78	− 2903.57	− 2815.16	0.00048	0.0764	0.1199
R2	ReQoL-UI	PHQ-9, GAD-7, age, sex	1340	3	28	0.8185	0.5772	0.9691	1512.10	− 2968.21	− 2822.59	0.00020	0.0763	0.1203
**R3**	**ReQoL-UI**	**PHQ-9, GAD-7, age, sex**	1340	4	39	0.8186	0.5855	0.9745	**1534.46**	**− 2990.93**	− 2788.11	**0.00006**	0.0758	0.1199
R4	ReQoL-UI	PHQ-9, GAD-7, sex	1340	2	16	0.8183	0.5440	0.9487	1468.71	− 2905.41	− 2822.21	0.00035	0.0763	0.1199
R5	ReQoL-UI	PHQ-9, GAD-7, sex	1340	3	26	0.8189	0.5960	0.9670	1509.95	− 2967.91	− 2832.70	− 0.00019	0.0760	0.1203
R6*	**ReQoL-UI**	**PHQ-9, GAD-7, sex**	1340	4	36	0.8185	0.1790	0.9429	1510.45	− 2948.90	− 2761.68	0.00018	**0.0751**	**0.1179**
R7	ReQoL-UI	PHQ-9, age, sex	1340	2	16	0.8185	0.5397	0.9488	1468.47	− 2904.95	− 2821.74	0.00020	0.0762	0.1198
R8	ReQoL-UI	PHQ-9, age, sex	1340	3	26	0.8189	0.5840	0.9683	1509.11	− 2966.23	− 2831.02	− 0.00019	0.0761	0.1202
R9	ReQoL-UI	PHQ-9, age, sex	1340	4	36	0.8189	0.5480	0.9453	1511.52	− 2951.03	− 2763.82	− 0.00021	0.0761	0.1199
R10	ReQoL-UI	PHQ-9, sex	1340	2	15	0.8185	0.5413	0.9488	1468.46	− 2906.91	− 2828.91	0.00014	0.0762	0.1197
R11	ReQoL-UI	PHQ-9, sex	1340	3	24	0.8193	0.5931	0.9664	1507.50	− 2967.00	**− 2842.19**	− 0.00067	0.0758	0.1202
R12	ReQoL-UI	PHQ-9, sex	1340	4	33	0.8196	0.6049	0.9665	1518.07	− 2970.14	− 2798.53	− 0.00093	0.0757	0.1204
V1	EQ-5D-5L VSE	PHQ-9, GAD-7, age, sex	1339	2	17	0.7904	0.5257	0.9490	884.23	− 1734.45	− 1646.06	− 0.00030	0.0953	0.1358
V2	EQ-5D-5L VSE	PHQ-9, GAD-7, age, sex	1339	3	28	0.7895	0.5441	0.9603	950.47	− 1844.94	− 1699.34	0.00061	0.0957	0.1358
V3*	**EQ-5D-5L VSE**	**PHQ-9, GAD-7, age, sex**	1339	4	39	0.7916	0.5142	0.9441	1045.47	− 2012.93	− 1810.15	− 0.00145	0.0954	**0.1352**
V4	EQ-5D-5L VSE	PHQ-9, GAD-7, age	1339	2	16	0.7905	0.5229	0.9485	883.72	− 1735.43	− 1652.24	− 0.00033	0.0953	0.1359
V5	EQ-5D-5L VSE	PHQ-9, GAD-7, age	1339	3	26	0.7896	0.5413	0.9535	947.63	− 1843.25	− 1708.06	0.00050	0.0958	0.1358
V6	EQ-5D-5L VSE	PHQ-9, GAD-7, age	1339	4	36	0.7917	0.5141	0.9458	1045.19	− 2018.38	− 1831.19	− 0.00153	0.0954	0.1352
V7	EQ-5D-5L VSE	PHQ-9, GAD-7, sex	1339	2	16	0.7906	0.5105	0.9486	882.88	− 1733.76	− 1650.57	− 0.00048	**0.0952**	0.1359
V8	EQ-5D-5L VSE	PHQ-9, GAD-7, sex	1339	3	26	0.7898	0.5229	0.9560	946.36	− 1840.73	− 1705.53	0.00035	0.0956	0.1359
V9	EQ-5D-5L VSE	PHQ-9, GAD-7, sex	1339	4	36	0.7897	0.5023	0.9581	993.18	− 1914.35	− 1727.16	0.00048	0.0955	0.1357
V10	EQ-5D-5L VSE	PHQ-9, GAD-7	1339	2	15	0.7907	0.5082	0.9480	882.39	− 1734.77	− 1656.78	− 0.00052	0.0953	0.1359
V11	EQ-5D-5L VSE	PHQ-9, GAD-7	1339	3	24	0.7898	0.5213	0.9491	944.31	− 1840.63	− 1715.83	**0.00029**	0.0957	0.1359
**V12**	**EQ-5D-5L VSE**	**PHQ-9, GAD-7**	1339	4	33	0.7909	0.5215	0.9437	**1048.00**	**− 2030.00**	**− 1858.41**	− 0.00073	0.0955	0.1357
C1	EQ-5D-5L Cross-walk	PHQ-9, GAD-7, age, sex	1339	2	17	0.7228	0.3882	0.9175	628.06	− 1222.13	− 1133.73	− 0.00171	0.1218	0.1664
C2	EQ-5D-5L Cross-walk	PHQ-9, GAD-7, age, sex	1339	3	28	0.7210	0.4132	0.9090	762.41	− 1468.82	− 1323.23	**0.00004**	0.1221	0.1661
C3*	**EQ-5D-5L Cross-walk**	**PHQ-9, GAD-7, age, sex**	1339	4	39	0.7220	0.3616	0.9320	**832.56**	**− 1587.13**	− 1384.34	− 0.00087	0.1218	**0.1657**
C4	EQ-5D-5L Cross-walk	PHQ-9, GAD-7, age	1339	2	16	0.7228	0.3860	0.9174	627.56	− 1223.11	− 1139.92	− 0.00169	0.1218	0.1664
C5	EQ-5D-5L Cross-walk	PHQ-9, GAD-7, age	1339	3	26	0.7211	0.4180	0.9154	759.38	− 1466.77	− 1331.57	− 0.00006	0.1223	0.1663
C6	EQ-5D-5L Cross-walk	PHQ-9, GAD-7, age	1339	4	36	0.7215	0.3945	0.9540	811.37	− 1550.75	− 1363.56	− 0.00041	0.1222	0.1664
C7	EQ-5D-5L Cross-walk	PHQ-9, GAD-7, sex	1339	2	16	0.7230	0.3671	0.9179	626.19	− 1220.38	− 1137.19	− 0.00193	0.1218	0.1664
C8	EQ-5D-5L Cross-walk	PHQ-9, GAD-7, sex	1339	3	26	0.7213	0.4074	0.9157	754.15	− 1456.29	− 1321.10	− 0.00025	0.1223	0.1664
**C9**	**EQ-5D-5L Cross-walk**	**PHQ-9, GAD-7, sex**	1339	4	36	0.7224	0.3577	0.9348	828.92	− 1585.85	− 1398.66	− 0.00133	**0.1217**	0.1658
C10	EQ-5D-5L Cross-walk	PHQ-9, GAD-7	1339	2	15	0.7230	0.3652	0.9176	625.62	− 1221.25	− 1143.25	− 0.00193	0.1219	0.1665
C11	EQ-5D-5L Cross-walk	PHQ-9, GAD-7	1339	3	24	0.7215	0.4055	0.9191	751.93	− 1455.86	− 1331.06	− 0.00043	0.1225	0.1665
C12	EQ-5D-5L Cross-walk	PHQ-9, GAD-7	1339	4	33	0.7225	0.3568	0.9311	821.69	− 1577.39	**− 1405.80**	− 0.00145	0.1220	0.1660

FAll models used the same number of observations (N = 1340 or 1339) and the same within component variables (Xvars): PHQ-9, GAD-7, age, sex. The best performing model specification within each performance statistic (i.e. LL, AIC, BIC, AE, MAE, and RMSE) is highlighted using bold font; the model number (Model No) is also highlighted in bold font in this instance; the final chosen model is marked with *

*Variable types*: PHQ-9, GAD-7, and age were classed as continuous variables; sex was classed as a binary variable

*AE* absolute error; *AIC* Akaike information criteria; *BIC* Bayesian information criteria; *DF* degrees of freedom; EQ-5D-5L, EQ-5D five-level version; *GAD*-7 generalised anxiety disorder-7; *LL* log likelihood; *MAE* mean absolute error; *PHQ*-9, patient health questionnatire-9; *ReQoL-UI* recovering quality of life – utility index; *RMSE* root mean square error; *VSE* value set for England

Model fit statistics for both ALDVMM and Betamix model specifications to the USVS are presented in Table 2. Although the ALDVMM fit statistics seemed reasonable, graphical methods identified an issue that suggested Betamix might be preferable (see “Comparison of mean predicted and observed utility scores” section). When using ALDVMMs and Betamix, both sets of models had convergence problems or were tending to unbounded models when attempting to fit 4-components; therefore, no 4-component model results are reported related to the USVS.

**Table 2:** Model fit statistics for the ALDVMMs or Betamix for the EQ-5D-5L USVS

Model No	Target	P-var	Obs	LC	DF	Mean	Min	Max	LL	AIC	BIC	AE	MAE	RMSE
ALDVMM**s**														
A-U1	EQ-5D-5L USVS	PHQ-9, GAD-7	1339	2	11	0.7581	0.4525	0.9388	585.18	− 1148.36	− 1091.16	− 0.00047	**0.1219**	0.1736
**A-U2**	**EQ-5D-5L USVS**	**PHQ-9, GAD-7**	1339	3	18	0.7577	0.4538	0.9353	**844.93**	− **1653.85**	− **1560.26**	− **0.00004**	0.1221	**0.1735**
A-U3	EQ-5D-5L USVS	PHQ-9, GAD-7	1339	4	25	–	–	–	–	–	–	–	–	–
Betamix														
B-U1*	**EQ-5D-5L USVS**	**PHQ-9, GAD-7**	1339	2	17	0.7576	0.4297	0.9549	402.98	− 771.97	− 683.57	**0.00003**	**0.1217**	**0.1735**
B-U2	EQ-5D-5L USVS	PHQ-9, GAD-7	1339	3	24	0.7568	0.4605	0.9503	**432.79**	− **817.58**	− **692.79**	0.00088	0.1224	0.1738
B-U3	EQ-5D-5L USVS	PHQ-9, GAD-7	1339	4	31	–	–	–	–	–	–	–	–	–

All models used the same number of observations (N = 1340 or 1339) and the same within component variables (Xvars): PHQ-9, GAD-7. The best performing model specification within each performance statistic (i.e. LL, AIC, BIC, AE, MAE, and RMSE) is highlighted using bold font; the model number (Model No) is also highlighted in bold font in this instance; the final chosen model is marked with *

*Variable types*: PHQ-9 and GAD-7 were classed as continuous variables

*AE* absolute error; *AIC* Akaike information criteria; *BIC* Bayesian information criteria; *DF* degrees of freedom; EQ-5D-5L, EQ-5D five-level version; *GAD*-7 generalised anxiety disorder-7; *LL* log likelihood; *MAE* mean absolute error; *PHQ*-9, patient health questionnatire-9; *ReQoL-UI* recovering quality of life – utility index; *RMSE* root mean square error; *VSE* value set for England

#### ReQoL-UI

The lowest predictive errors (i.e. lowest MAE and RMSE values) were attained when the pvars were PHQ-9, GAD-7, and sex (e.g. model R6). Including age as an additional pvar increased goodness of fit (i.e. higher LL and lower AIC values); however, it does so by increasing the predictive error (i.e. increased RMSE and MAE values) for example when comparing between R3 and R6. The lowest BIC was for R11 which is not surprising given the way BIC penalises having more variables, despite the benefits the inclusion of more variables has on performance statistics other than BIC such as for R3 and R6.

#### EQ-5D-5L VSE

The lowest RMSE value was obtained when the pvars were PHQ-9, GAD-7, age, sex (i.e. V3), but goodness of fit improved when age and sex were not included as pvars (i.e. V12). The lowest MAE was for V7 which was a 2-component model which did not include age as a pvar; however, moving from a 2- to 4-component model tended to improve goodness of fit and RMSE, at the cost of MAE.

#### EQ-5D-5L Cross-walk

The best goodness of fit statistics and RMSE were when the pvars were PHQ-9, GAD-7, age, and sex (i.e. C3). BIC was lowest for the model with the least pvars (similar to the ReQoL-UI and VSE); the lowest MAE was for C9.

#### EQ-5D-5L USVS

Betamix was preferred to ALDVMMs. For the Betamix models, the lowest predictive error was for a 2-component model; although, the better goodness of fit statistics were for the 3-component model.

### Comparison of mean predicted and observed utility scores

**Fig. 2:**
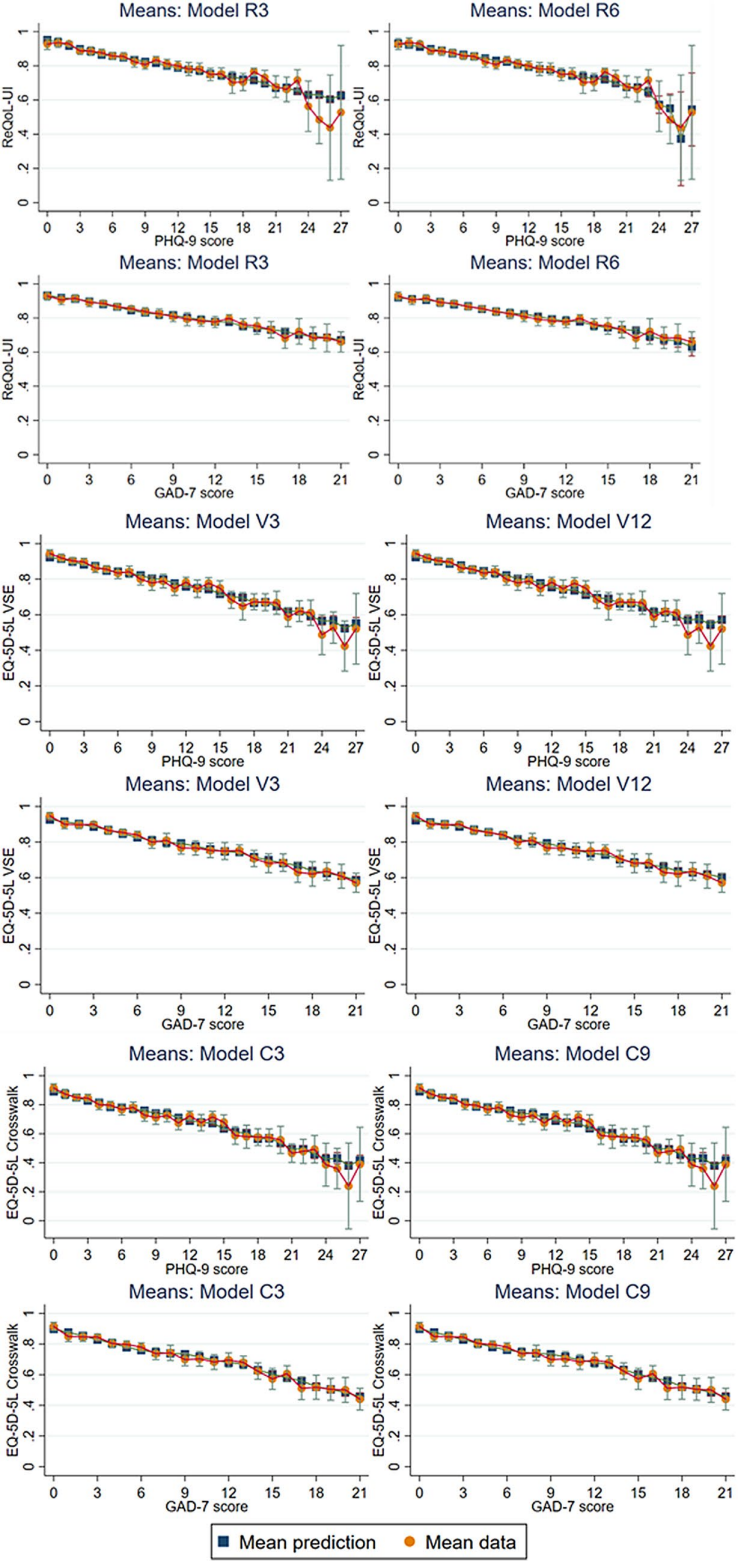
Mean predicted and observed utility scores for models: R3 Vs R6; V3 Vs V12; C3 Vs C9

**Fig. 3:**
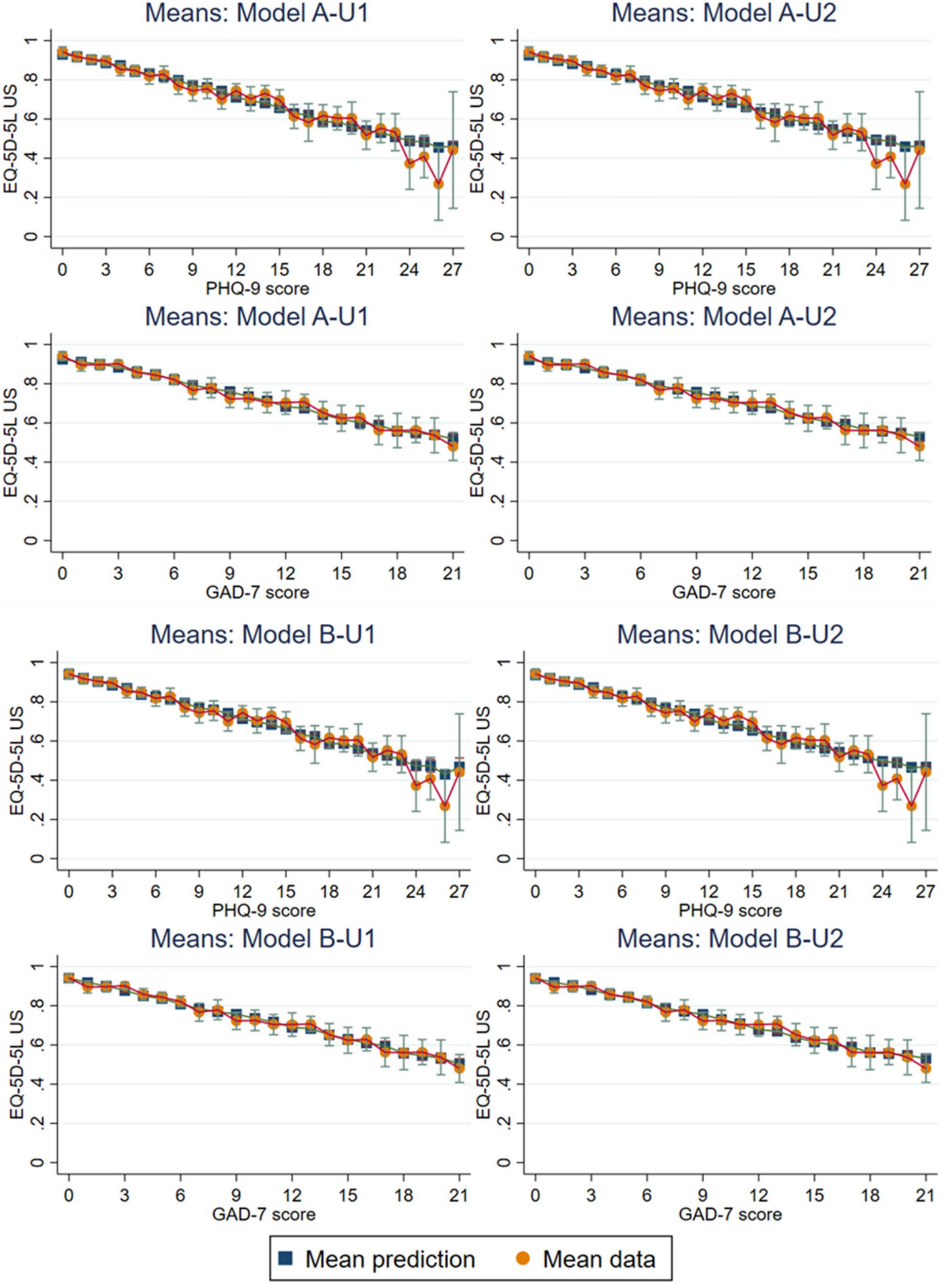
Mean predicted and observed utility scores for ALDVMMs (A-U1 Vs A-U2) and Betamix models (B-U1 Vs B-U2)

**Fig. 4:**
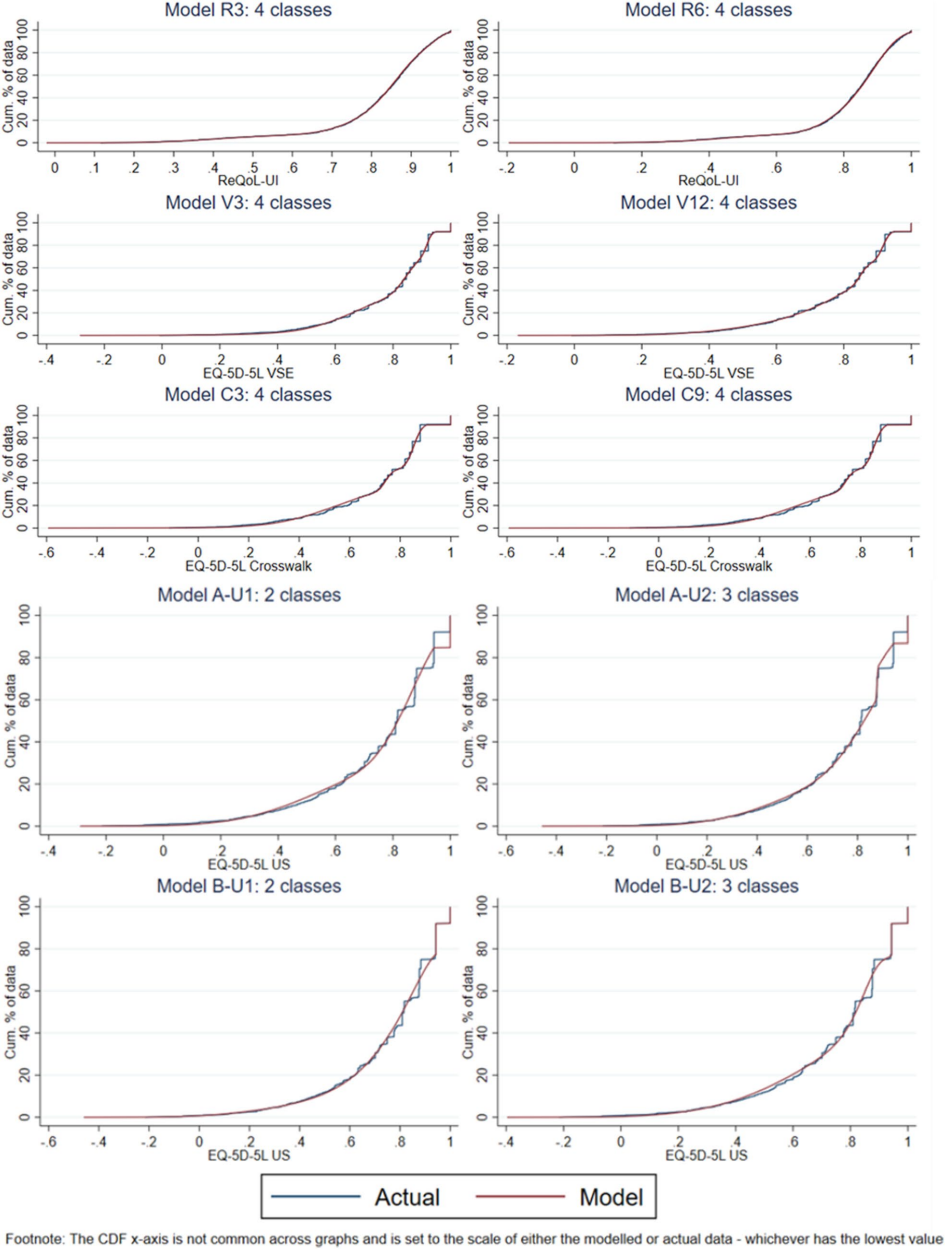
Cumulative distribution functions for the simulated data for models: R3 Vs R6; V3 Vs V12; C3 Vs C9; A-U1 Vs A-U2; B-U1 Vs B-U2

Based on model fit statistics, we use graphical methods to compare between the following 4-component models: R3 and R6; V3 and V12; C3 and C9. For the USVS, we use graphical methods to compare between 2- and 3-component, ALDVMM (A-U1 Vs A-U2) and Betamix (B-U1 Vs B-U2) models. Figure 2 (UK/England utility scores) and Fig. 3 (USVS) presents the mean predicted and observed utility scores, and Fig. 4 presents the CDFs for the simulated data.

#### ReQoL-UI

The benefits of R6’s lower MAE and RMSE relative to R3 becomes more apparent in Fig. 2, particularly based on the observed versus predicted utility scores at the severe end of the PHQ-9 score scale i.e. ≥ 23. That is, we can visually see that the predicted error for R3 is larger than for R6 for those people with a PHQ-9 score ≥ 23. Across the GAD-7 score scale, the predicted errors seems visually similar between models R3 and R6. Based on the CDFs there is little difference between the actual and modelled data for both R3 and R6, so this suggests both models fit equally well in terms of the distribution.

#### EQ-5D-5L VSE

The visual comparison between V3 and V12 is less clear-cut than between R3 and R6. Figure 2 indicates both models map well across the GAD-7 score scale, but have larger predictive errors at the severe end of the PHQ-9 score scale i.e. ≥ 23. Although not instantly obvious based on the CDFs (Fig. 4), V3 does fit slightly better than V12 across the utility score range of 0.6 to 0.9.

#### EQ-5D-5L Cross-walk

The visual comparison between C3 and C9 is again less clear-cut, with Fig. 2 again suggesting good fit with the GAD-7, larger predictive error when PHQ-9 score scale ≥ 23, and almost identical CDFs; this is not surprising though given the almost identical model fit statistics with small between-model trade-offs in MAE and RMSE.

#### EQ-5D-5L USVS

Although the mapping function from the ALDVMMs fit reasonably across the clinical (Fig. 3) and utility score range (Fig. 4), the models were not fitting well for higher utility values; such that the proportion of perfect health values (1) implied by the estimated ALDVMMs is too high, as shown in Fig. 4. In comparison, the Betamix models overcame this issue with lower predictive error statistics than for the ALDVMMs, also shown in Fig. 4. Figure 4 visual comparisons between B-U1 and B-U2 revealed a slightly better fit across the middle score range (e.g. between 0.4 and 0.7) with similar fit across the rest of the score range.

### Choosing a mapping function

For each target UK/England utility score, comparisons were made across all 12 models; however, for descriptive purposes, here we focus just on comparisons between models: R3 and R6; V3 and V12; C3 and C9.

- ReQoL-UI: R6 is chosen due to its lower MAE and RMSE, but also based on the visual comparisons across the mean predicted and observed utility scores across the PHQ-9 and GAD-7 score ranges.
- EQ-5D-5L VSE: V3 is chosen due to its lower MAE and RMSE despite the differences between models not initially being visually obvious using graphical methods.
- EQ-5D-5L Cross-walk: C3 is chosen due to its lower RMSE and better goodness of fit statistics; although, the model was very similar to C9 both in terms of model fit statistics and based on graphical methods.
  For the USVS when comparing between the 2-component and 3-component Betamix models, the predictive error statistics and fit through visual inspection was better for the 2-component model despite the 3-component model having the better AIC and BIC. Therefore:
- EQ-5D-5L USVS: B-U1 was chosen because of its fit at higher utility scores than the ALDVMMs, and lower predictive errors both in statistics and visually compared to the other Betamix model (B-U2).

## Discussion

Across all mapping models to UK/England utility scores, we selected 4-component models where utility within each component was a function of PHQ-9, GAD-7, age, and sex. For mapping to the ReQoL-UI we selected R6, where the probability of component membership was a function of PHQ-9, GAD-7, and sex. For mapping to the EQ-5D-5L VSE or cross-walk we selected V3 or C3, respectively, where the probability of component membership was a function of PHQ-9, GAD-7, sex, and age. Results pertaining to alternative model specifications are presented in Appendix S2.

For the USVS, the mapping process and results were more complicated. For the ALDVMMs, the models did not fit well for higher utility values, such that the proportion of perfect health values (1) implied by the estimated model was too high. Even though moving from 2- to 3-components reduced the proportion of ones, ALDVMMs were unable to match the observed proportion. The problem stemmed from the large probability mass present in the USVS sample distribution just below the gap (see Fig. 1) which would require a degenerate distribution. This is difficult to achieve with the ALDVMM, thus leading to the decision to use Betamix that is able to generate a separate probability mass at the truncation point.

Predictions from our recommended mapping functions are provided in an Excel-based lookup table, provided as part of the online Supplementary Materials.

### Mapping to the USVS relative to the UK/England utility scores

The USVS in our estimation sample caused complications for our identified ALDVMMs that did not occur when mapping to the EQ-5D-5L VSE or cross-walk, nor ReQoL-UI. It should be noted that ALDVMMs are quicker and easier to fit than Betamix; however, Betamix has been developed to have more modelling options and therefore some additional flexibility for mapping than ALDVMMs when required. In this case, it was the ability of Betamix to specify probability mass at the upper (i.e. 1) and truncation (i.e. 0.943) values of the USVS which enabled us to overcome the problems when using ALDVMMs at the upper end of the utility scale, despite the additional computational time and considerations required to fit Betamix relative to ALDVMMs.

### Comparisons with previous mapping studies

We identified three previous mapping studies relevant for comparison with our mapping study from the GAD-7 and/or PHQ-9 to the ReQoL-UI and/or EQ-5D (five or three-level versions) as part our pre-mapping considerations to inform our mapping plans.

Brazier et al. [16] included the GAD-7 and PHQ-9 (among other mental health measures) with intentions to map to the EQ-5D-3L and SF-6D. This study used more traditional mapping models (OLS, Tobit, and response-level) rather than more modern and currently recommended mixture models; however, Brazier et al. [16] was published in 2014 before mapping using mixture models gained widespread attention. It is important to note that Brazier et al. [16] never mapped from the GAD-7 and PHQ-9 to the EQ-5D(-3L); rather, they mapped from the GAD-7 and PHQ-9 only to the SF-6D, with an alternative mental health measure (the Hospital Anxiety and Depression Scale, HADS) being used to map to the EQ-5D-3L. This was because the IAPT estimation dataset (one of four datasets) they had available with the PHQ-9 and GAD-7 only included the SF-6D, not the EQ-5D-3L. However, through inference from all the mapping they conducted, their overall conclusion was that “mapping from mental health condition-specific measures, such as the widely used PHQ-9, GAD and HADS, may not be an appropriate approach to generating EQ-5D and SF-6D scores as these measures focus on specific symptoms and not on the wider impact of mental health conditions”. Our current mapping study and associated previous psychometric analysis does not concur with Brazier et al. [16] conclusion [24], noting that our mapping studies are not completely alike (e.g. due to using a different target measure). However, reasons our conclusions do not concur could be associated with our use of more suitable mixture regression models for mapping compared to traditional mapping models (e.g. OLS) which have known limitations, that we are using the newer EQ-5D-5L rather than the previous EQ-5D-3L which has known shortcomings in mental health populations, and that we mapped from the PHQ-9 and GAD-7 to the EQ-5D-5L (and ReQoL-UI) which this previous study did not [13–20, 25–27].

Furukawa et al. [55] ‘mapped’ from the PHQ-9 to the EQ-5D-3L using a non-regression-based approach (i.e. equipercentile linking); however, Furukawa et al. [55] does not describe itself as a mapping study and thus does not follow any current mapping guidance. The current first author published a correspondence about the study by Furukawa et al. [55] which outlines concerns about the study and the ‘mapping function’ it produced, to which a response was also published [53, 54]. Overall, the study by Furukawa et al. [55] provides little to no model performance statistics, thus comparisons cannot be made with our current mapping study.

Keetharuth and Rowen [60], a non-peer-reviewed article, mapped from the HoNOS to the ReQoL-UI. Although Keetharuth and Rowen [60] follow mapping guidance and is appropriately reported, it has two key limitations: first, only OLS models are used; second, the HoNOS is clinician-reported thus the completer’s perspective is different to that of the ReQoL-UI (i.e. patient-reported) which limits the conceptual overlap between the two measures. Keetharuth and Rowen [60] recognise these limitations, thus recommend caution when using their mapping functions.

Overall, previous mapping studies have not produced mapping functions between our source and target measures, with those mapping studies which are somewhat comparable to ours using more traditional regression-based (e.g. OLS) or non-regression-based (i.e. equipercentile linking) methods compared to the more modern and currently recommended mixture regression models we have used. Our study further emphasises the benefits of using mixture models, with ALDVMMs being a good starting point as they work well for mapping when used appropriately [25–27]. Alternatively, Betamix can overcome the shortcomings of ALDVMMs (e.g. for the USVS in our study), noting Betamix is computationally more complicated and time consuming despite its relative benefits, thus ALDVMMs are the preferred starting model as was the case for this study. Overall, our mapping functions represent a needed tool for predicting utility values from the commonly used PHQ-9 and GAD-7 mental health measures.

### Using the alternative predictions: aspects for consideration

Although all our predicted utility scores can be used to estimate QALYs, the source of these utility scores requires careful consideration. Firstly, each of our target utility scores have been shown to produce different QALYs [23]; therefore, it is logical to assume these predictions will produce different QALYs. The EQ-5D-5L is the more commonly used and known preference-based measure, relative to the newer ReQoL-UI. The constructs of these measures are different; although both are suggested to be ‘generic health measures’, the descriptive system of EQ-5D-5L is more physical health focussed relative to the ReQoL-UI’s more mental health focus. The measures and associated utility scores have also been shown to have different relationships with anxiety and depression as measured by the GAD-7 and PHQ-9, respectively, which will have influenced the mapping models [24]

### Use of predicted utility scores: strengths and limitations

The mapping predictions have been estimated from a specific patient population involved in an IAPT-based trial: new IAPT Step 2 service referrals who met the trial eligibility criteria. IAPT Step 2 focusses on specific mental health populations and interventions; i.e. common mental health conditions that could benefit from low intensity therapies as brief psychological interventions (e.g. digital mental health interventions, Bibliotherapy) offered with support from clinicians [61]. Additionally, our data collection time-period covers a 12-months care pathway when the patient is on a waiting-list or treatment, and a period during post-discharge. As such, we have less data that covers the ‘severe’ spectrum of anxiety and depression (mainly from baseline assessment) and this could explain our mapping models’ poorer performance at the severe end of the scale. Therefore, in mental health populations where ‘severe’ depression and anxiety is more prevalent (e.g. inpatient settings), our mapping functions are prone to higher predictive errors; alternative mapping predictions should be sought in such severe patient populations. For mental health trials wanting to use the predictions, consideration should be given to how an IAPT Step 2 population is representative of their trial population; for example, comparative assessment against our PROM score distributions in Fig. 1 with additional estimation sample descriptive statistics in Appendix S1.

## Conclusion

Our mapping functions can be used to predict either the ReQoL-UI, EQ-5D-5L VSE, USVS or cross-walked utility scores from the PHQ-9 and GAD-7 summary scores. Our analyses found that including more than one component improved model fit, with the preferred ALDVMMs based on 4-component models, and that Betamix was preferred to ALDVMMs when mapping to the USVS only. Our mapping functions can be used in economic evaluations to predict utility as a function of the commonly collected PHQ-9 and/or GAD-7 summary scores.

## Supplementary Information

Below is the link to the electronic supplementary material.

Supplementary file1 (DOCX 1525 KB)

Supplementary file2 (XLS 24634 KB)

## Data Availability

The data used for analysis is available on reasonable request to SilverCloud Health; please contact Derek Richards or Angel Enrique.
