# Supplementary material for: Enabling QALY estimation in mental health trials and care settings: mapping from the PHQ-9 and GAD-7 to the ReQoL-UI or EQ-5D-5L using mixture models
Source: Qual Life Res. 2023 Jun 14;32(10):2763–78. doi: 10.1007/s11136-023-03443-9 (PMC10474206; doi:10.1007/s11136-023-03443-9)
Supplement: Supplementary file 1 — Supplementary file1 (DOCX 1525 KB) [file 11136_2023_3443_MOESM1_ESM.docx]

**Full title: Enabling QALY estimation in mental health trials and care settings: mapping from the PHQ-9 and GAD-7 to the ReQoL-UI or EQ-5D-5L using mixture models**

**Short title: Mapping PHQ-9 and GAD-7 to ReQoL-UI or EQ-5D-5L**

**Supplementary Appendices**

**Contents Page**

[Appendix S1: Additional methods and descriptive statistics 2](#_Toc115358880)

[Appendix S2: Mapping from the PHQ-9 or GAD-7 to the ReQoL-UI or EQ-5D-5L UK value sets 5](#_Toc115358881)

# **Appendix S1: Additional methods and descriptive statistics**

**Patient-reported outcome measures (PROMs).** Table S1.1 provides a summarised overview of all PROMs used for analysis, including the Recovering Quality of Life – Utility Index (ReQoL-UI), EQ-5D five-level version (EQ-5D-5L), Patient Health Questionnaire-9 (PHQ-9), and Generalized Anxiety Disorder-7 (GAD-7).

**Estimation data source: trial eligibility criteria.** Trial inclusion criteria were people: (i) aged between 18-80 years; (ii) above clinical thresholds for depression (PHQ-9 ≥ 10) or anxiety (GAD-7 ≥ 8) (1-3), and (iii) suitable for iCBT (i.e. willing to use iCBT, internet access). Exclusion criteria included: suicidal ideation/intended (PHQ-9 question 9 score > 2 and/or during clinical interview); psychotic illness; organic mental health disorder; alcohol and/or drug misuse; and currently receiving psychological treatment.

**Moving from 2- to 3- and 4- component models.** Fitting 2-component models tends to be relatively simple based on the *aldvmm* or *betamix* command and maximum likelihood estimation within Stata. However, fitting 3- and 4- component models can lead to unbounded solutions (whereby maximum likelihood estimates become artificially high) and convergence problems, therefore, some previous users of these commands have suggested it is not possible to move beyond 2- to 3-components. Here we first fitted 2-component models based on the various combinations of xvars and pvars that could be specified (i.e. GAD-7, PHQ-9, age, and sex), then we assessed each 2-component model’s LL, AIC, and BIC to decide which model specifications to attempt fitting 3- and 4-components. As such, we identified that including xvars of age and sex tended to improve model fit, as did including both the GAD-7 and PHQ-9 as xvars; therefore, all models reported here are based on the following xvars: GAD-7, PHQ-9, age, and sex. Models based on xvars of PHQ-9 or GAD-7 independently (with age and sex) rather than together in one model, are reported in Appendix S2. Therefore, for descriptive purposes, in the main manuscript we focussed on models with the same xvars but pre-assessed pvar specification per target utility score with 2-4 components

**Model fit statistics and the need for graphical methods**. Standard measures based on “errors” (difference between the data and the model prediction) often provide conflicting results because they are based on different scoring functions. For example, RMSE penalizes the existence of large outliers more than MAE. Both AIC and BIC are likelihood-based criteria with a penalty for model complexity, but the penalty BIC imposes tends to be larger, often resulting in AIC and BIC selecting models with different number of parameters. Also, the LL, and associated AIC and BIC, are not comparable between the ALDVMMs and Betamix models given that models based on the beta distribution need rescaling of the dependent variable to the range of the health state value set, invalidating comparisons of AIC and BIC (4). Because of these issues, graphical methods have been shown to be essential for mapping model selection (5).

**Estimation sample descriptive statistics.** Table S1.2 reports summary statistics of the sample at baseline and for the pooled sample along with the number of patients, N, by time-points. The PROM scores at baseline are lower than across all time-points with GAD-7 and PHQ-9 following the reverse pattern. This indicates an improvement of health for all measures across time.

**Table S1.1. Description of outcomes measures and associated scores**

| **Long name** | **Short Name** | **Construct** | **No. items** | **Item score** | **Scoring type** | **Floor/**  **worst** | **Ceiling/**  **best** | **Cut-offs and description** | **Ref** |
| --- | --- | --- | --- | --- | --- | --- | --- | --- | --- |
| Recovering Quality of Life – Utility Index | ReQoL-UI | Preference-based recovery-focussed quality of life in mental health service users | 7 | 5-point scale:  0 (worst state) to  4 (best state) | Preference-based value set for the UK | -0.195 | 1 | N/A | (6, 7) |
| EQ-5D five-level version | EQ-5D-5L | Preference-based generic health | 5 | 5-point scale:  1 (no problem) to  5 (extreme/unable) | Cross-walked to the EQ-5D-3L UK value set* | -0.594 | 1 | N/A | (8) |
|  |  |  |  |  | Preference-based value set for England (VSE) | -0.285 | 1 | N/A | (9) |
|  |  |  |  |  | Preference-based United States value set (USVS) | -0.573 | 1 | N/A | (10) |
| Patient Health Questionnaire-9 | PHQ-9 | Depression severity | 9 | 4-point scale:  0 (not at all) to  3 (nearly every day) | Summary | 27 | 0 | <10, No caseness;  ≥10, Caseness  OR  <5, Minimal;  5-9, Mild;  10-14, Moderate;  15-19, Moderately severe;  ≥20, Severe | (3, 11) |
| Generalized Anxiety Disorder-7 | GAD-7 | Anxiety severity | 7 | 4-point scale:  0 (not at all) to  3 (nearly every day) | Summary | 21 | 0 | <8, No caseness;  ≥8, Caseness  OR  <5, Minimal;  5-9, Mild;  10-14, Moderate;  ≥15, Severe | (1, 2, 11) |

**Table S1.2. Descriptive statistics of estimation sample at baseline and across the six data collection points**

|  | **Baseline (N = 353^a^)** | | | | **All time-points (Obs = 1340 ^a^)** | | | |
| --- | --- | --- | --- | --- | --- | --- | --- | --- |
|  | **Mean** | **SD** | **Min** | **Max** | **Mean** | **SD** | **Min** | **Max** |
| ReQoL-UI | 0.778 | 0.141 | 0.115 | 0.995 | 0.819 | 0.145 | 0.115 | 1 |
| EQ-5D-5L cross-walk | 0.653 | 0.202 | 0.076^b^ | 1 | 0.721 | 0.206 | -0.116^b^ | 1 |
| EQ-5D-5L VSE | 0.731 | 0.162 | -0.010 | 1 | 0.790 | 0.168 | -0.010 | 1 |
| EQ-5D-5L USVS | 0.684 | 0.201 | -0.216 | 1 | 0.758 | 0.213 | -0.216 | 1 |
| PHQ-9 | 14.4 | 5.0 | 2 | 27 | 9.7 | 6.3 | 0 | 27 |
| GAD-7 | 12.6 | 4.6 | 0 | 21 | 8.8 | 5.7 | 0 | 21 |
| Age | 33.0 | 12.3 | 18 | 74 | - | - | - | - |
|  |  |  |  |  |  |  |  |  |
| Female, n (%) | 251 (71.1%) | - | - | - | - | - | - | - |

**Acronyms.** GAD-7, generalised anxiety disorder-7; PHQ-9, patient health questionnatire-9; ReQoL-UI, recovering quality of life – utility index; SD, standard deviation; USVS, United States value set; VSE, Value set for England

^a^ N that completed the ReQoL-UI, EQ-5D-5L PHQ-9, and GAD-7 by time-point: baseline, 353; 8-week, 283; 3-month, 183; 6-month, 179; 9-month, 173; 12-month, 169. However, there was one less observation for the EQ-5D-5L than the other patient-reported measures, so for the EQ-5D-5L: baseline, N = 352; all time-points, Obs = 1339.

^b^ Across all preference-based scores, the min value is a score at baseline thus why the min score at ‘Baseline’ and ‘All time-points’ is the same value; the only exception is for the EQ-5D-5L cross-walk. For the EQ-5D-5L cross-walk the min value at baseline (0.076) is different to across all time-points for which the min value (-0.116) is for a different person as reported at 12-months. This is because of the preference-based score associated with the worst reported EQ-5D-5L health profile at baseline (43444) relative to an alternative health state profile only reported at 12-months (33454). The preference-scores for health profiles 43444 and 33454, respectively, are as follows:

cross-walk, 0.076 vs. -0.116; VSE, -0.01 vs. 0.062; USVS, -0.216 vs. -0.197. Only the cross-walk assigns a worse preference-based scores to health profile 33454 relative to 43444, whereas the VSE and USVS assigns a worse preference-based score to 43444 relative to 33454. As such, this is why the min value at ‘Baseline’ is different to ‘All time-points’ for the EQ-5D-5L cross-walk only, because of the relative preference-based score assigned specifically to the health profile of 43444 compared to 33454.

# **Appendix S2: Mapping from the PHQ-9 or GAD-7 to the ReQoL-UI or EQ-5D-5L UK value sets**

**Objective.** To describe mapping from the GAD-7 and/or PHQ-9 to the ReQoL-UI or EQ-5D-5L using alternative xvar (and associated pvar) specifications to those described in the main manuscript. For example, the GAD-7 but not the PHQ-9 is part of one specification set described here; therefore, if a target (e.g. trial) dataset has GAD-7 but not PHQ-9 data, one of the mapping functions described here is more appropriate to use than that in the main manuscript which requires both the PHQ-9 and GAD-7 to be available in the target dataset.

**Methods.** The mapping methods are the same as described in the main manuscript. The key difference is the specification of the xvars and pvars, with the choice of pvars limited to the set of covariates chosen for the xvar set; for example, the choice of xvars are:

- **Xvar set 1:** PHQ-9 summary score, age, and sex;
- **Xvar set 2:** GAD-7 summary score, age, and sex;
- **Xvar set 3:** PHQ-9 and GAD-7 summary score.

**Model fit statistics.** Model fit statistics for xvar sets 1, 2, and 3 are presented in Tables S1.1, S1.2, and S1.3, respectively. In Table S1.1 there were two LC4 models with very low probabilities which fitted some outliers, which was deemed not viable/useful for mapping purposes and therefore the results are omitted from the table.

**Comparison of mean predicted and observed utility scores.** For xvar sets 1, 2, and 3, Figures 1.1, 1.3, and 1.5 presents the mean predicted and observed utility scores, and Figures 1.2, 1.4, and 1.6 presents the CDFs for the simulated data, respectively. Based on model fit statistics, we used these graphical methods to compare between the following 4-component models:

- **Xvar set 1:** P-R9 and P-R12; P-V9 and P-V12; P-C3 and P-C6.
- **Xvar set 2:** G-R3 and G-R9; G-V3 and G-V9; G-C3 and G-C6.
- **Xvar set 3:** PG-R3 and PG-R9; PG-V3 and PG-V6; PG-C3 and PG-C6.

**Choosing a mapping function.** For each target utility score, comparisons were made across all models reported within Tables S1.1, 1.2, or 1.3.

***ReQoL-UI***

- **Xvar set 1**: **P-R12** was chosen because of its lower AIC, MAE and RMSE, and after visual inspection across the mean predicted and observed utility score, and CDFs.
- **Xvar set 2**: **G-R3** was chosen due its lower MAE and RMSE, despite visually being very similar to the other suggested mapping function (i.e. G-R9).
- **Xvar set 3**: **PG-R9** was chosen because of its lower AIC and MAE, and after visual inspection across the mean predicted and observed utility scores, and CDFs.

***EQ-5D-5L VSE***

- **Xvar set 1**: **P-V12** was chosen because of its lower AIC, BIC, and RMSE, and after visual inspection across the mean predicted and observed utility scores, and CDFs.
- **Xvar set 2**: **G-V9** was chosen because of its lower AE, MAE, and RMSE, and after visual inspection across the mean predicted and observed utility scores, and CDFs.
- **Xvar set 3**: **PG-V6** was chosen because of its lower MAE and RMSE, and after visual inspection across the mean predicted and observed utility scores, and CDFs.

***EQ-5D-5L Crosswalk***

- **Xvar set 1**: **P-C3** was chosen because of its lower AIC and RMSE, and after visual inspection across the mean predicted and observed utility scores, and CDFs.
- **Xvar set 2**: G-C3 was chosen due to its lower RMSE and higher overall LL, despite visually and statistically being very similar to the other suggested mapping function (i.e. G-C6).
- **Xvar set 3**: **PG-C6** was chosen because of its lower MAE and RMSE, and after visual inspection across the mean predicted and observed utility scores, and CDFs.

**Conclusion.** Our mapping functions can be used to predict either the ReQoL-UI, EQ-5D-5L VSE or cross-walked utility scores from the PHQ-9 and/or GAD-7 summary scores dependent on the available covariates in the target dataset.

**Table S2.1. Model fit statistics for the ALDVMMs for the ReQoL-UI, EQ-5D-5L VSE and cross-walk when the xvars are PHQ-9, age, and sex**

| **Model No.** | **Target** | **P-var** | **Obs.** | **LC** | **DF** | **Mean** | **Min** | **Max** | **LL** | **AIC** | **BIC** | **AE** | **MAE** | **RMSE** |
| --- | --- | --- | --- | --- | --- | --- | --- | --- | --- | --- | --- | --- | --- | --- |
| P-R1 | ReQoL-UI | PHQ-9, age, sex | 1340 | 2 | 14 | 0.8182 | 0.5504 | 0.9448 | 1454.05 | -2880.11 | -2807.30 | 0.00050 | 0.0768 | 0.1206 |
| P-R2 | ReQoL-UI | PHQ-9, age, sex | 1340 | 3 | 23 | 0.8185 | 0.5869 | 0.9655 | 1496.28 | -2946.55 | -2826.95 | 0.00019 | 0.0766 | 0.1208 |
| P-R3 | ReQoL-UI | PHQ-9, age, sex | 1340 | 4 | - | - | - | - | - | - | - | - | - | - |
| P-R4 | ReQoL-UI | PHQ-9, sex | 1340 | 2 | 13 | 0.8184 | 0.5609 | 0.9441 | 1453.56 | -2881.11 | -2813.51 | 0.00025 | 0.0767 | 0.1206 |
| P-R5 | ReQoL-UI | PHQ-9, sex | 1340 | 3 | 21 | 0.8188 | 0.6044 | 0.9624 | 1493.75 | -2945.50 | -2836.29 | **-0.00010** | 0.0765 | 0.1210 |
| P-R6 | ReQoL-UI | PHQ-9, sex | 1340 | 4 | - | - | - | - | - | - | - | - | - | - |
| P-R7 | ReQoL-UI | PHQ-9, age | 1340 | 2 | 13 | 0.8178 | 0.5599 | 0.9413 | 1451.06 | -2876.12 | -2808.52 | 0.00088 | 0.0772 | 0.1208 |
| P-R8 | ReQoL-UI | PHQ-9, age | 1340 | 3 | 21 | 0.8183 | 0.5950 | 0.9629 | 1494.38 | -2946.76 | -2837.55 | 0.00041 | 0.0769 | 0.1210 |
| **P-R9** | **ReQoL-UI** | **PHQ-9, age** | 1340 | 4 | 29 | 0.8182 | 0.5673 | 0.9500 | **1501.68** | -2945.36 | -2794.55 | 0.00046 | 0.0770 | 0.1207 |
| P-R10 | ReQoL-UI | PHQ-9 | 1340 | 2 | 12 | 0.8181 | 0.5742 | 0.9397 | 1450.23 | -2876.46 | -2814.05 | 0.00060 | 0.0770 | 0.1209 |
| P-R11 | ReQoL-UI | PHQ-9 | 1340 | 3 | 19 | 0.8185 | 0.6134 | 0.9589 | 1491.59 | -2945.19 | **-2846.38** | 0.00017 | 0.0769 | 0.1213 |
| **P-R12*** | **ReQoL-UI** | **PHQ-9** | 1340 | 4 | 26 | 0.8183 | 0.2815 | 0.9582 | 1501.47 | **-2950.94** | -2815.73 | 0.00036 | **0.0757** | **0.1190** |
|  |  |  |  |  |  |  |  |  |  |  |  |  |  |  |
| P-V1 | EQ-5D-5L VSE | PHQ-9, age, sex | 1339 | 2 | 14 | 0.7903 | 0.5285 | 0.9431 | 846.91 | -1665.81 | -1593.02 | -0.00020 | 0.0978 | 0.1391 |
| P-V2 | EQ-5D-5L VSE | PHQ-9, age, sex | 1339 | 3 | 23 | 0.7903 | 0.5172 | 0.9457 | 896.45 | -1746.89 | -1627.30 | **-0.00014** | 0.0977 | 0.1387 |
| P-V3 | EQ-5D-5L VSE | PHQ-9, age, sex | 1339 | 4 | 32 | 0.7897 | 0.5282 | 0.9527 | 928.22 | -1792.43 | -1626.05 | 0.00048 | 0.0983 | 0.1388 |
| P-V4 | EQ-5D-5L VSE | PHQ-9, sex | 1339 | 2 | 13 | 0.7905 | 0.5197 | 0.9422 | 846.02 | -1666.05 | -1598.45 | -0.00036 | 0.0977 | 0.1390 |
| P-V5 | EQ-5D-5L VSE | PHQ-9, sex | 1339 | 3 | 21 | 0.7905 | 0.5116 | 0.9394 | 929.52 | -1817.05 | -1707.85 | -0.00040 | **0.0977** | 0.1390 |
| P-V6 | EQ-5D-5L VSE | PHQ-9, sex | 1339 | 4 | 29 | 0.7898 | 0.5214 | 0.9482 | 924.65 | -1791.29 | -1640.50 | 0.00034 | 0.0982 | 0.1389 |
| P-V7 | EQ-5D-5L VSE | PHQ-9, age | 1339 | 2 | 13 | 0.7904 | 0.5258 | 0.9421 | 846.18 | -1666.37 | -1598.77 | -0.00026 | 0.0978 | 0.1391 |
| P-V8 | EQ-5D-5L VSE | PHQ-9, age | 1339 | 3 | 21 | 0.7903 | 0.5220 | 0.9403 | 931.61 | -1821.21 | -1712.02 | -0.00021 | 0.0977 | 0.1391 |
| **P-V9** | **EQ-5D-5L VSE** | **PHQ-9, age** | 1339 | 4 | 29 | 0.7909 | 0.5089 | 0.9444 | **993.52** | -1929.04 | -1778.25 | -0.00080 | 0.0979 | 0.1385 |
| P-V10 | EQ-5D-5L VSE | PHQ-9 | 1339 | 2 | 12 | 0.7906 | 0.5174 | 0.9411 | 845.31 | -1666.62 | -1604.22 | -0.00043 | 0.0978 | 0.1391 |
| P-V11 | EQ-5D-5L VSE | PHQ-9 | 1339 | 3 | 19 | 0.7906 | 0.5094 | 0.9470 | 894.09 | -1750.17 | -1651.38 | -0.00045 | 0.0977 | 0.1388 |
| **P-V12*** | **EQ-5D-5L VSE** | **PHQ-9** | 1339 | 4 | 26 | 0.7911 | 0.5044 | 0.9463 | 991.37 | **-1930.73** | **-1795.54** | -0.00095 | 0.0978 | **0.1385** |
|  |  |  |  |  |  |  |  |  |  |  |  |  |  |  |
| P-C1 | EQ-5D-5L Cross-walk | PHQ-9, age, sex | 1339 | 2 | 14 | 0.7227 | 0.3911 | 0.9110 | 592.87 | -1157.74 | -1084.94 | -0.00159 | 0.1240 | 0.1707 |
| P-C2 | EQ-5D-5L Cross-walk | PHQ-9, age, sex | 1339 | 3 | 23 | 0.7209 | 0.4135 | 0.9036 | 716.95 | -1387.90 | -1268.31 | 0.00014 | 0.1244 | 0.1704 |
| **P-C3*** | **EQ-5D-5L Cross-walk** | **PHQ-9, age, sex** | 1339 | 4 | 32 | 0.7215 | 0.3420 | 0.9310 | **786.05** | **-1508.10** | -1341.71 | -0.00043 | 0.1238 | **0.1695** |
| P-C4 | EQ-5D-5L Cross-walk | PHQ-9, sex | 1339 | 2 | 13 | 0.7228 | 0.3782 | 0.9106 | 591.70 | -1157.40 | -1089.80 | -0.00177 | 0.1240 | 0.1707 |
| P-C5 | EQ-5D-5L Cross-walk | PHQ-9, sex | 1339 | 3 | 21 | 0.7214 | 0.3918 | 0.9052 | 711.79 | -1381.58 | -1272.38 | -0.00029 | 0.1244 | 0.1706 |
| **P-C6** | **EQ-5D-5L Cross-walk** | **PHQ-9, sex** | 1339 | 4 | 29 | 0.7219 | 0.3407 | 0.9322 | 782.49 | -1506.98 | -1356.19 | -0.00079 | **0.1237** | 0.1696 |
| P-C7 | EQ-5D-5L Cross-walk | PHQ-9, age | 1339 | 2 | 13 | 0.7227 | 0.3885 | 0.9103 | 592.07 | -1158.13 | -1090.54 | -0.00160 | 0.1241 | 0.1708 |
| P-C8 | EQ-5D-5L Cross-walk | PHQ-9, age | 1339 | 3 | 21 | 0.7212 | 0.4093 | 0.9064 | 714.78 | -1387.57 | -1278.37 | **-0.00008** | 0.1245 | 0.1706 |
| P-C9 | EQ-5D-5L Cross-walk | PHQ-9, age | 1339 | 4 | 29 | 0.7217 | 0.3387 | 0.9229 | 776.99 | -1495.98 | -1345.19 | -0.00065 | 0.1241 | 0.1698 |
| P-C10 | EQ-5D-5L Cross-walk | PHQ-9 | 1339 | 2 | 12 | 0.7229 | 0.3760 | 0.9096 | 590.84 | -1157.67 | -1095.28 | -0.00180 | 0.1241 | 0.1708 |
| P-C11 | EQ-5D-5L Cross-walk | PHQ-9 | 1339 | 3 | 19 | 0.7217 | 0.3928 | 0.9080 | 709.59 | -1381.18 | -1282.38 | -0.00057 | 0.1245 | 0.1707 |
| P-C12 | EQ-5D-5L Cross-walk | PHQ-9 | 1339 | 4 | 26 | 0.7221 | 0.3386 | 0.9223 | 773.31 | -1494.63 | **-1359.44** | -0.00103 | 0.1239 | 0.1698 |

**Footnote.** All models used the same number of observations (N = 1339) and the same within component variables (Xvars): PHQ-9, age, sex. The best performing model specification within each performance statistic (i.e. LL, AIC, BIC, AE, MAE, and RMSE) is highlighted using **bold** font; the model number (Model No) is also highlighted in **bold** font in this instance; the final chosen model is marked with *.

**Variable types.** PHQ-9 and age were classed as continuous variables; sex was classed as a binary variable.

**Acronyms.** AE, absolute error; AIC, Akaike information criteria; BIC, Bayesian information criteria; DF, degrees of freedom; EQ-5D-5L, EQ-5D five-level version; GAD-7, generalised anxiety disorder-7; LL, log likelihood; MAE, mean absolute error; PHQ-9, patient health questionnatire-9; ReQoL-UI, recovering quality of life – utility index; RMSE, root mean square error; VSE, value set for England.

**
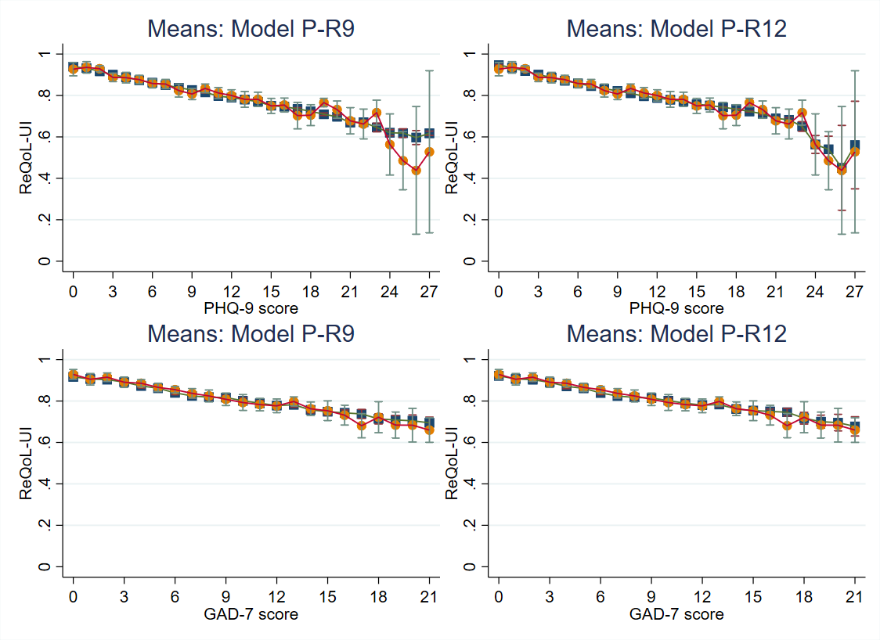
**

**
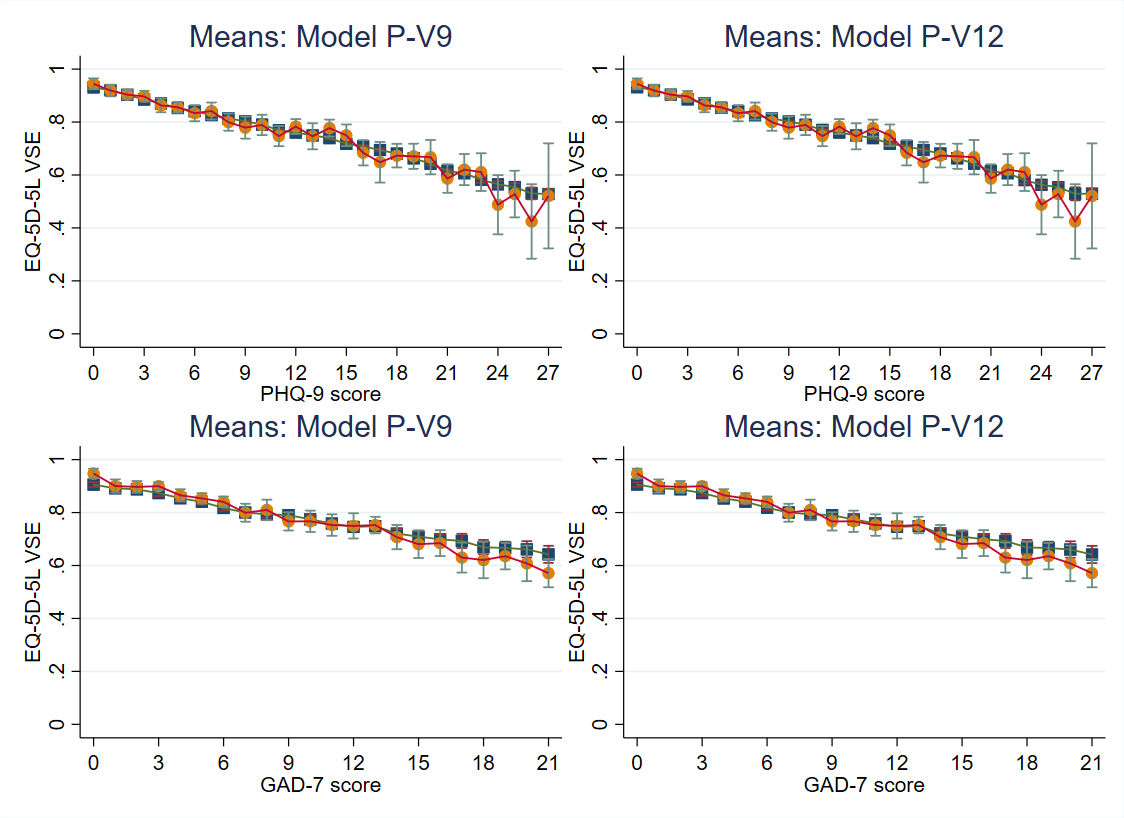
**

**
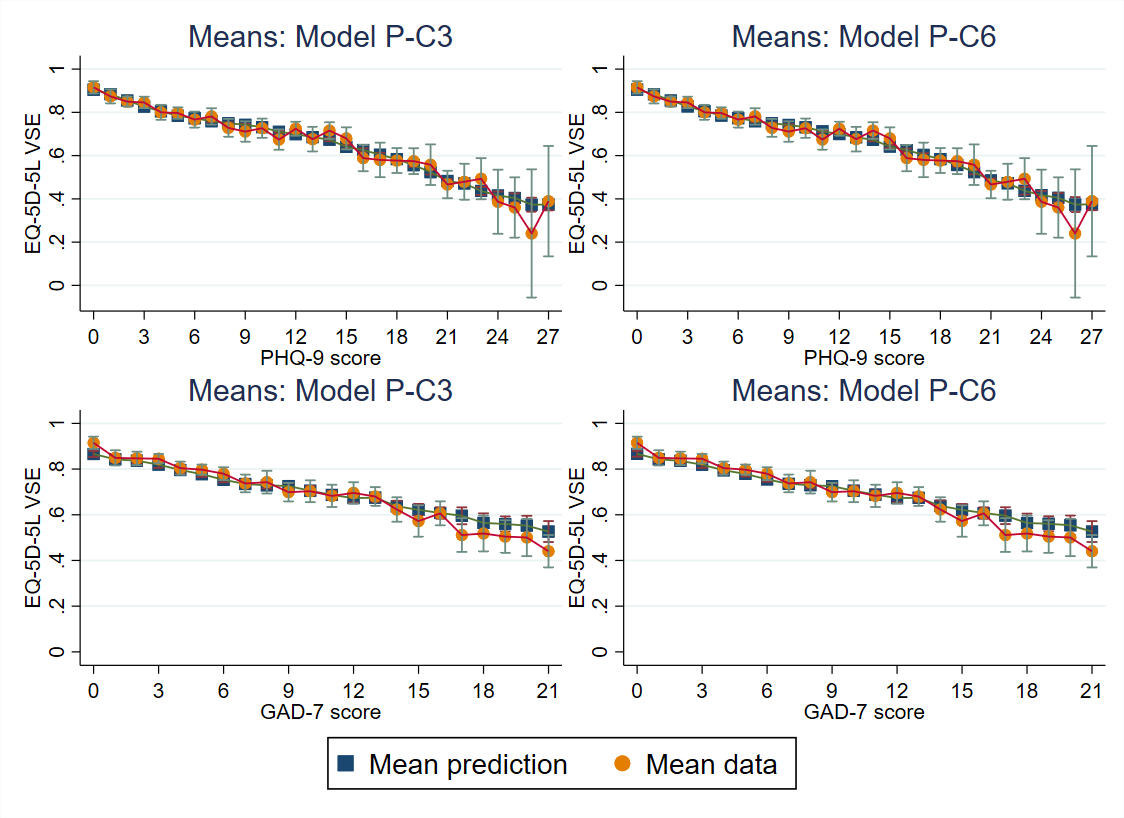
**

**Figure S2.1. Mean predicted and observed utility scores for P-models: P-R9 Vs P-R12; P-V9 Vs P-V12; P-C3 Vs P-C6**


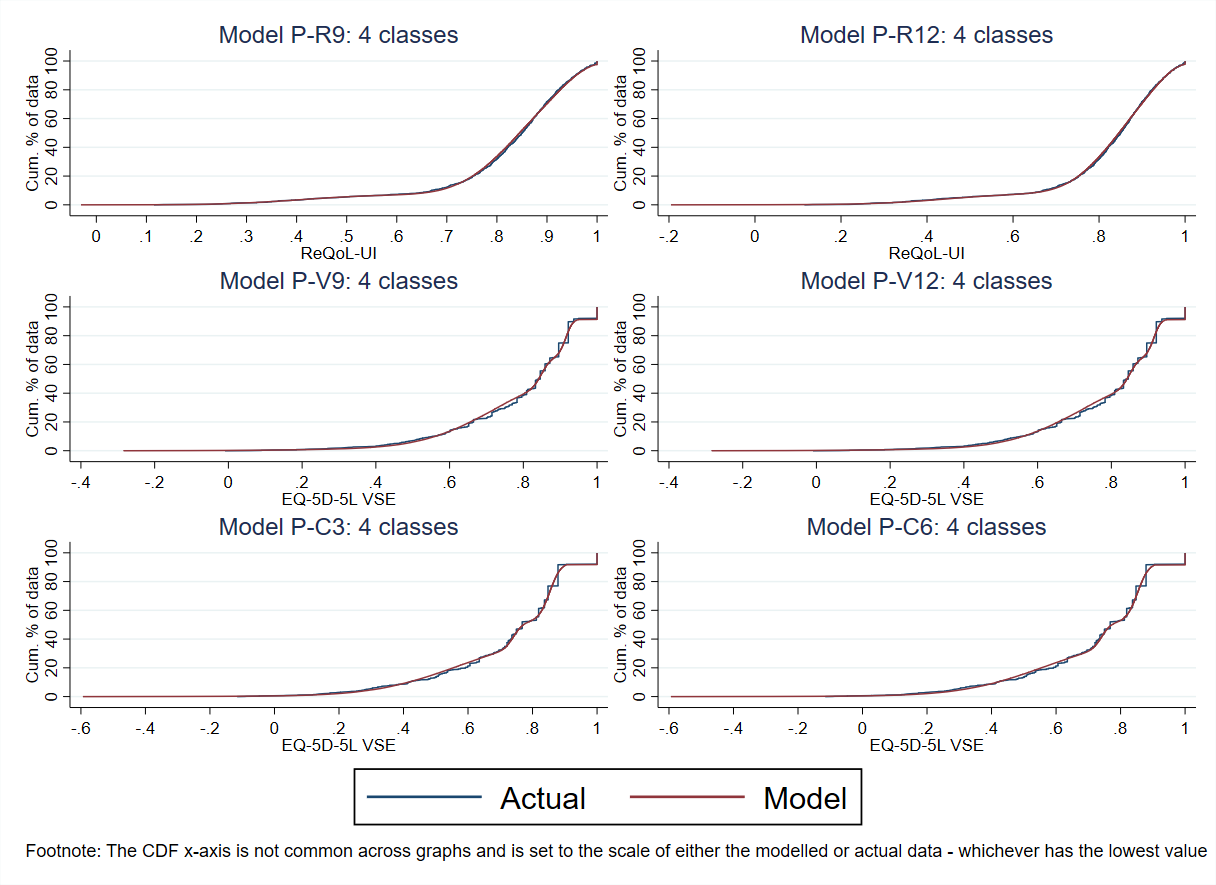


**Figure S2.2. Cumulative distribution functions for the simulated data for P-models: P-R9 Vs P-R12; P-V9 Vs P-V12; P-C3 Vs P-C6**

**Table S2.2. Model fit statistics for the ALDVMMs for the ReQoL-UI, EQ-5D-5L VSE and cross-walk when the xvars are GAD-7, age, and sex**

| **Model No.** | **Target** | **P-var** | **Obs.** | **LC** | **DF** | **Mean** | **Min** | **Max** | **LL** | **AIC** | **BIC** | **AE** | **MAE** | **RMSE** |
| --- | --- | --- | --- | --- | --- | --- | --- | --- | --- | --- | --- | --- | --- | --- |
| G-R1 | ReQoL-UI | GAD-7, age, sex | 1340 | 2 | 14 | 0.8181 | 0.5966 | 0.9274 | 1296.56 | -2565.12 | -2492.31 | 0.00059 | 0.0831 | 0.1261 |
| G-R2 | ReQoL-UI | GAD-7, age, sex | 1340 | 3 | 23 | 0.8184 | 0.6199 | 0.9544 | 1335.44 | -2624.87 | -2505.26 | 0.00028 | 0.0827 | 0.1260 |
| **G-R3*** | **ReQoL-UI** | **GAD-7, age, sex** | 1340 | 4 | 32 | 0.8184 | 0.4765 | 0.9515 | 1354.79 | -2645.57 | -2479.16 | 0.00023 | 0.0825 | **0.1257** |
| G-R4 | ReQoL-UI | GAD-7, sex | 1340 | 2 | 13 | 0.8182 | 0.6004 | 0.9273 | 1296.42 | -2566.84 | -2499.23 | 0.00045 | 0.0830 | 0.1261 |
| G-R5 | ReQoL-UI | GAD-7, sex | 1340 | 3 | 21 | 0.8188 | 0.6425 | 0.9528 | 1333.14 | -2624.28 | -2515.07 | -0.00017 | 0.0825 | 0.1261 |
| G-R6 | ReQoL-UI | GAD-7, sex | 1340 | 4 | 29 | 0.8189 | 0.5250 | 0.9510 | 1348.92 | -2639.85 | -2489.04 | -0.00026 | **0.0824** | 0.1258 |
| G-R7 | ReQoL-UI | GAD-7, age | 1340 | 2 | 13 | 0.8178 | 0.6035 | 0.9248 | 1290.26 | -2554.51 | -2486.91 | 0.00083 | 0.0834 | 0.1263 |
| G-R8 | ReQoL-UI | GAD-7, age | 1340 | 3 | 21 | 0.8182 | 0.6284 | 0.9507 | 1332.65 | -2623.29 | -2514.08 | 0.00046 | 0.0829 | 0.1261 |
| **G-R9** | **ReQoL-UI** | **GAD-7, age** | 1340 | 4 | 29 | 0.8184 | 0.6235 | 0.9556 | **1356.09** | **-2654.18** | -2503.37 | 0.00022 | 0.0827 | 0.1260 |
| G-R10 | ReQoL-UI | GAD-7 | 1340 | 2 | 12 | 0.8185 | 0.4424 | 0.9499 | 1290.13 | -2556.26 | -2493.85 | 0.00068 | 0.0834 | 0.1262 |
| G-R11 | ReQoL-UI | GAD-7 | 1340 | 3 | 19 | 0.8186 | 0.6550 | 0.9456 | 1329.73 | -2621.46 | **-2522.65** | **0.00004** | 0.0827 | 0.1262 |
| G-R12 | ReQoL-UI | GAD-7 | 1340 | 4 | 26 | 0.8178 | 0.6224 | 0.9456 | 1338.58 | -2625.16 | -2489.95 | 0.00084 | 0.0832 | 0.1263 |
|  |  |  |  |  |  |  |  |  |  |  |  |  |  |  |
| G-V1 | EQ-5D-5L VSE | GAD-7, age, sex | 1339 | 2 | 14 | 0.7904 | 0.5621 | 0.9345 | 825.76 | -1623.52 | -1550.72 | -0.00030 | 0.0999 | 0.1400 |
| G-V2 | EQ-5D-5L VSE | GAD-7, age, sex | 1339 | 3 | 23 | 0.7894 | 0.5763 | 0.9414 | 886.20 | -1726.40 | -1606.81 | 0.00069 | 0.1003 | 0.1400 |
| **G-V3** | **EQ-5D-5L VSE** | **GAD-7, age, sex** | 1339 | 4 | 32 | 0.7895 | 0.5573 | 0.9433 | **921.86** | -1779.71 | -1613.32 | 0.00061 | 0.1002 | 0.1399 |
| G-V4 | EQ-5D-5L VSE | GAD-7, sex | 1339 | 2 | 13 | 0.7906 | 0.5503 | 0.9340 | 824.74 | -1623.48 | -1555.88 | -0.00046 | 0.0998 | 0.1401 |
| G-V5 | EQ-5D-5L VSE | GAD-7, sex | 1339 | 3 | 21 | 0.7911 | 0.5650 | 0.9436 | 879.71 | -1717.42 | -1608.23 | -0.00093 | 0.0999 | 0.1395 |
| G-V6 | EQ-5D-5L VSE | GAD-7, sex | 1339 | 4 | 29 | 0.7896 | 0.5534 | 0.9398 | 920.66 | **-1783.31** | -1632.52 | 0.00050 | 0.1002 | 0.1400 |
| G-V7 | EQ-5D-5L VSE | GAD-7, age | 1339 | 2 | 13 | 0.7905 | 0.5598 | 0.9338 | 825.26 | -1624.52 | -1556.93 | -0.00032 | 0.0999 | 0.1401 |
| G-V8 | EQ-5D-5L VSE | GAD-7, age | 1339 | 3 | 21 | 0.7904 | 0.5943 | 0.9416 | 884.40 | -1726.80 | -1617.60 | -0.00026 | 0.1000 | 0.1397 |
| **G-V9*** | **EQ-5D-5L VSE** | **GAD-7, age** | 1339 | 4 | 29 | 0.7902 | 0.5886 | 0.9690 | 909.09 | -1760.17 | -1609.38 | **-0.00009** | **0.0995** | **0.1394** |
| G-V10 | EQ-5D-5L VSE | GAD-7 | 1339 | 2 | 12 | 0.7906 | 0.5484 | 0.9333 | 824.25 | -1624.50 | -1562.11 | -0.00048 | 0.0998 | 0.1402 |
| G-V11 | EQ-5D-5L VSE | GAD-7 | 1339 | 3 | 19 | 0.7913 | 0.5654 | 0.9481 | 878.61 | -1719.21 | -1620.42 | -0.00113 | 0.1000 | 0.1395 |
| G-V12 | EQ-5D-5L VSE | GAD-7 | 1339 | 4 | 26 | 0.7895 | 0.5470 | 0.9324 | 917.06 | -1782.12 | **-1646.93** | 0.00065 | 0.1003 | 0.1402 |
|  |  |  |  |  |  |  |  |  |  |  |  |  |  |  |
| G-C1 | EQ-5D-5L Cross-walk | GAD-7, age, sex | 1339 | 2 | 14 | 0.7227 | 0.4332 | 0.9002 | 581.17 | -1134.34 | -1061.54 | -0.00166 | **0.1261** | 0.1712 |
| G-C2 | EQ-5D-5L Cross-walk | GAD-7, age, sex | 1339 | 3 | 23 | 0.7211 | 0.4823 | 0.8960 | 703.97 | -1361.94 | -1242.35 | **0.00000** | 0.1268 | 0.1712 |
| **G-C3*** | **EQ-5D-5L Cross-walk** | **GAD-7, age, sex** | 1339 | 4 | 32 | 0.7216 | 0.4323 | 0.9234 | **766.84** | -1469.69 | -1303.30 | -0.00053 | 0.1263 | **0.1706** |
| G-C4 | EQ-5D-5L Cross-walk | GAD-7, sex | 1339 | 2 | 13 | 0.7229 | 0.4188 | 0.9004 | 579.86 | -1133.71 | -1066.12 | -0.00183 | 0.1261 | 0.1712 |
| G-C5 | EQ-5D-5L Cross-walk | GAD-7, sex | 1339 | 3 | 21 | 0.7213 | 0.4703 | 0.9004 | 697.38 | -1352.76 | -1243.56 | -0.00022 | 0.1269 | 0.1713 |
| **G-C6** | **EQ-5D-5L Cross-walk** | **GAD-7, sex** | 1339 | 4 | 29 | 0.7220 | 0.4309 | 0.9272 | 764.33 | **-1470.65** | -1319.86 | -0.00090 | 0.1262 | 0.1707 |
| G-C7 | EQ-5D-5L Cross-walk | GAD-7, age | 1339 | 2 | 13 | 0.7227 | 0.4308 | 0.8999 | 580.72 | -1135.44 | -1067.84 | -0.00164 | 0.1261 | 0.1712 |
| G-C8 | EQ-5D-5L Cross-walk | GAD-7, age | 1339 | 3 | 21 | 0.7211 | 0.4972 | 0.9012 | 700.81 | -1359.62 | -1250.43 | -0.00006 | 0.1269 | 0.1714 |
| G-C9 | EQ-5D-5L Cross-walk | GAD-7, age | 1339 | 4 | 29 | 0.7215 | 0.4681 | 0.9322 | 760.84 | -1463.68 | -1312.89 | -0.00046 | 0.1267 | 0.1713 |
| G-C10 | EQ-5D-5L Cross-walk | GAD-7 | 1339 | 2 | 12 | 0.7229 | 0.4173 | 0.8999 | 579.37 | -1134.74 | -1072.34 | -0.00182 | 0.1261 | 0.1713 |
| G-C11 | EQ-5D-5L Cross-walk | GAD-7 | 1339 | 3 | 19 | 0.7215 | 0.4650 | 0.9042 | 695.21 | -1352.41 | -1253.62 | -0.00037 | 0.1269 | 0.1714 |
| G-C12 | EQ-5D-5L Cross-walk | GAD-7 | 1339 | 4 | 26 | 0.7218 | 0.4645 | 0.9332 | 758.38 | -1464.77 | **-1329.58** | -0.00076 | 0.1267 | 0.1714 |

**Footnote.** All models used the same number of observations (N = 1340 or 1339) and the same within component variables (Xvars): GAD-7, age, sex. The best performing model specification within each performance statistic (i.e. LL, AIC, BIC, AE, MAE, and RMSE) is highlighted using **bold** font; the model number (Model No) is also highlighted in **bold** font in this instance.

**Variable types.** GAD-7 and age were classed as continuous variables; sex was classed as a binary variable.

**Acronyms.** AE, absolute error; AIC, Akaike information criteria; BIC, Bayesian information criteria; DF, degrees of freedom; EQ-5D-5L, EQ-5D five-level version; GAD-7, generalised anxiety disorder-7; LL, log likelihood; MAE, mean absolute error; PHQ-9, patient health questionnatire-9; ReQoL-UI, recovering quality of life – utility index; RMSE, root mean square error; VSE, value set for England.

**
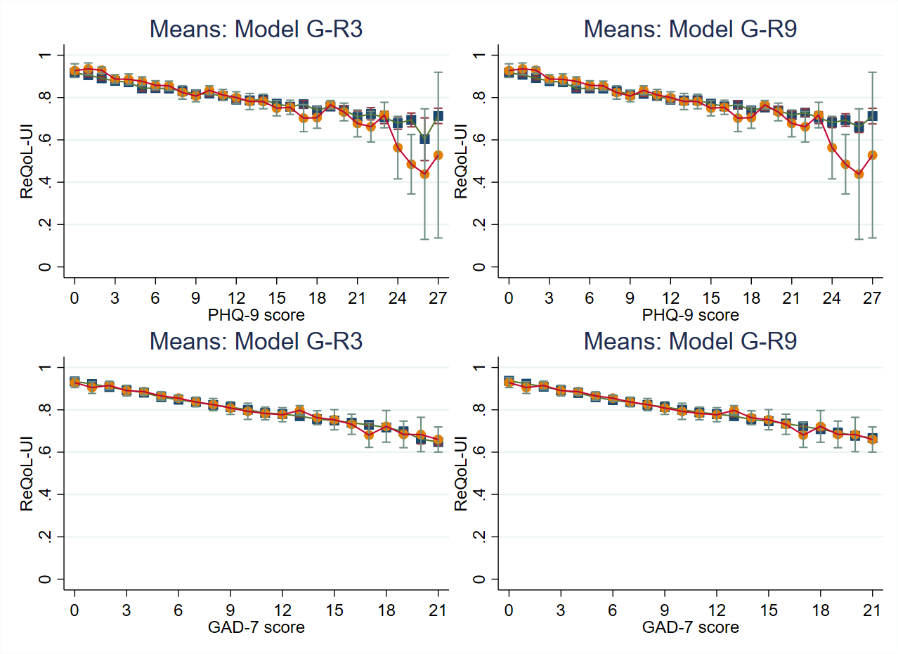
**

**
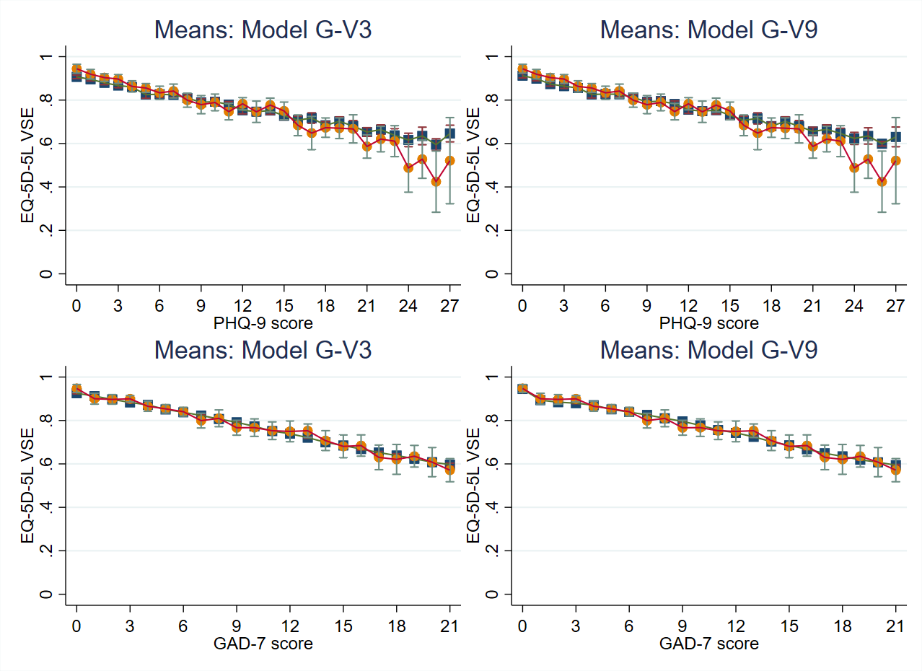
**

**
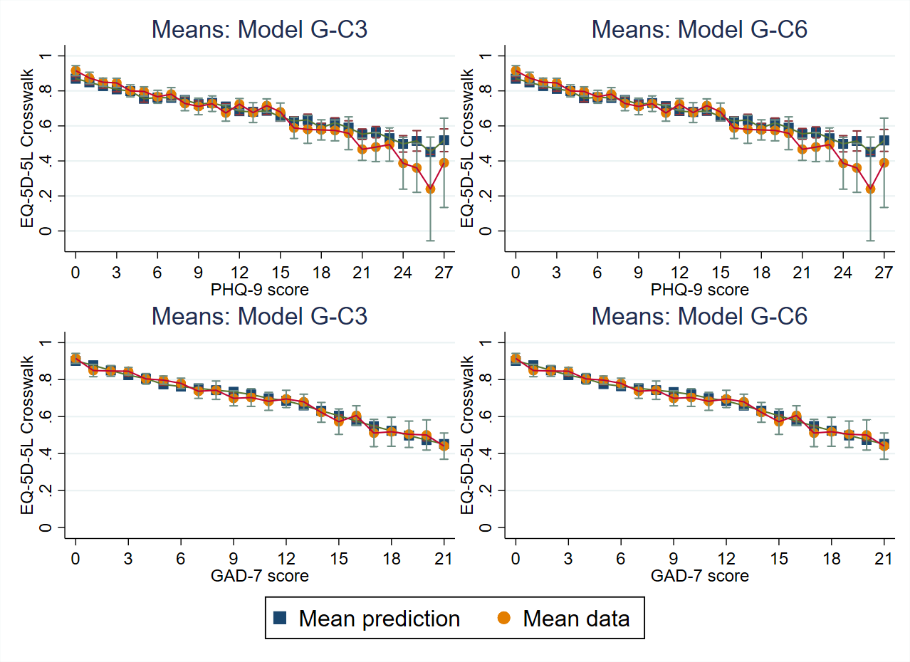
**

**Figure S2.3. Mean predicted and observed utility scores for G-models: G-R3 Vs G-R9; G-V3 Vs G-V9; G-C3 Vs G-C6**

**
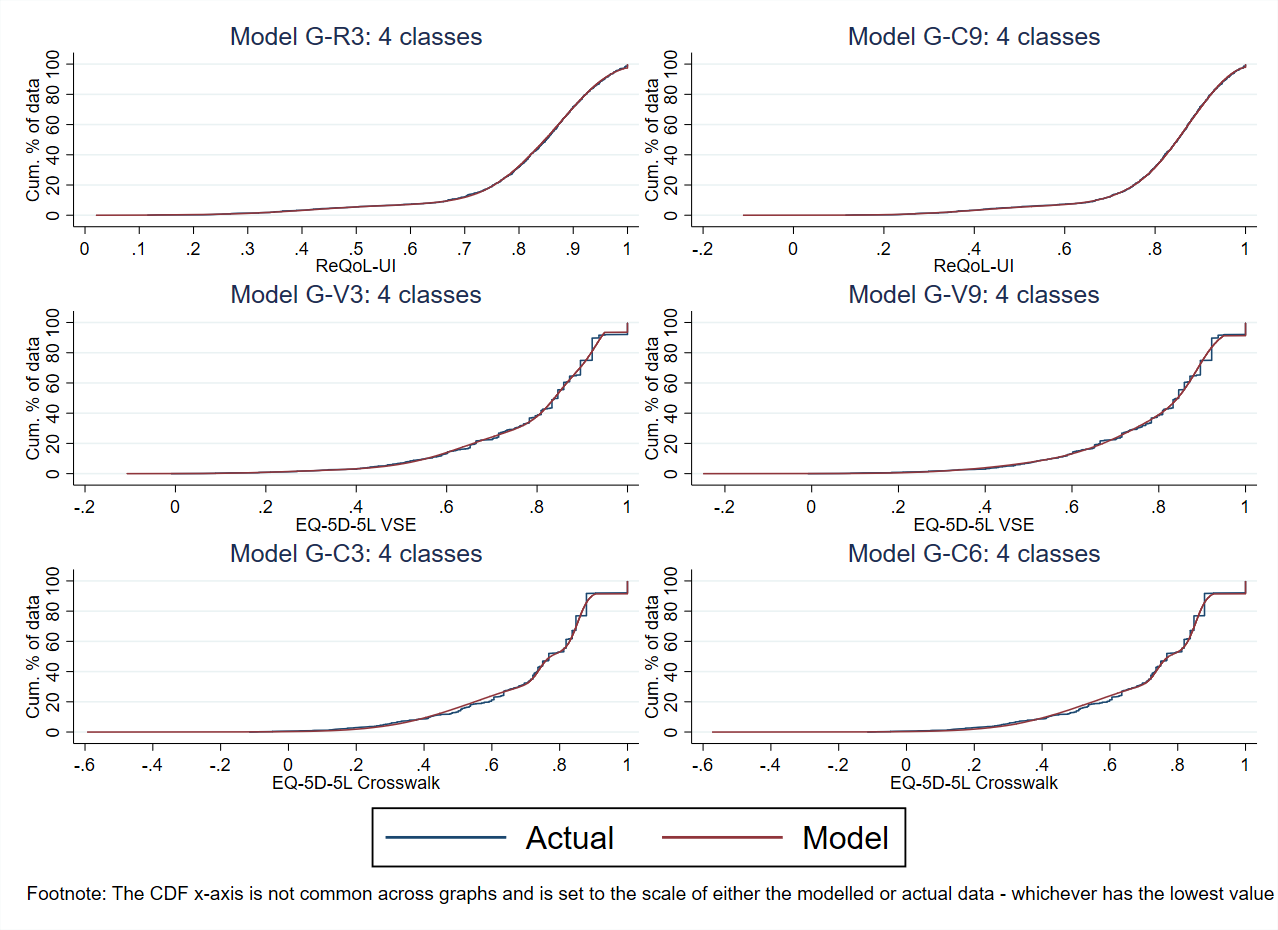
**

**Figure S2.4. Cumulative distribution functions for the simulated data for G-models: G-R3 Vs G-R9; G-V3 Vs G-V9; G-C3 Vs G-C6**

**Table S2.3. Model fit statistics for the ALDVMMs for the ReQoL-UI, EQ-5D-5L VSE and cross-walk when the xvars are PHQ-9 and GAD-7**

| **Model No.** | **Target** | **P-var** | **Obs.** | **LC** | **DF** | **Mean** | **Min** | **Max** | **LL** | **AIC** | **BIC** | **AE** | **MAE** | **RMSE** |
| --- | --- | --- | --- | --- | --- | --- | --- | --- | --- | --- | --- | --- | --- | --- |
| PG-R1 | ReQoL-UI | PHQ-9, GAD-7 | 1340 | 2 | 11 | 0.8181 | 0.5902 | 0.9342 | 1446.71 | -2871.41 | -2814.21 | 0.00053 | 0.0770 | **0.1209** |
| PG-R2 | ReQoL-UI | PHQ-9, GAD-7 | 1340 | 3 | 18 | 0.8185 | 0.6237 | 0.9533 | 1490.34 | -2944.68 | -2851.07 | 0.00021 | 0.0769 | 0.1213 |
| **PG-R3** | **ReQoL-UI** | **PHQ-9, GAD-7** | 1340 | 4 | 25 | 0.8185 | 0.6229 | 0.9540 | **1504.52** | -2959.04 | -2829.03 | 0.00018 | 0.0768 | 0.1212 |
| PG-R4 | ReQoL-UI | PHQ-9 | 1340 | 2 | 10 | 0.8185 | 0.5886 | 0.9331 | 1444.65 | -2869.30 | -2817.30 | 0.00014 | 0.0769 | 0.1209 |
| PG-R5 | ReQoL-UI | PHQ-9 | 1340 | 3 | 16 | 0.8185 | 0.6219 | 0.9520 | 1486.78 | -2941.56 | **-2858.35** | 0.00014 | 0.0770 | 0.1215 |
| PG-R6 | ReQoL-UI | PHQ-9 | 1340 | 4 | 22 | 0.8186 | 0.6125 | 0.9541 | 1500.78 | -2957.57 | -2843.16 | 0.00005 | 0.0768 | 0.1214 |
| PG-R7 | ReQoL-UI | GAD-7 | 1340 | 2 | 10 | 0.8181 | 0.6066 | 0.9333 | 1445.48 | -2870.97 | -2818.96 | 0.00056 | 0.0771 | 0.1211 |
| PG-R8 | ReQoL-UI | GAD-7 | 1340 | 3 | 16 | 0.8185 | 0.6174 | 0.9445 | 1485.00 | -2938.01 | -2854.80 | 0.00013 | 0.0769 | 0.1211 |
| **PG-R9*** | **ReQoL-UI** | **GAD-7** | 1340 | 4 | 22 | 0.8185 | 0.6112 | 0.9509 | 1501.55 | **-2959.10** | -2844.69 | 0.00014 | **0.0767** | 0.1211 |
|  |  |  |  |  |  |  |  |  |  |  |  |  |  |  |
| PG-V1 | EQ-5D-5L VSE | PHQ-9, GAD-7 | 1339 | 2 | 11 | 0.7905 | 0.5537 | 0.9307 | 869.33 | -1716.66 | -1659.47 | -0.00032 | 0.0966 | 0.1368 |
| PG-V2 | EQ-5D-5L VSE | PHQ-9, GAD-7 | 1339 | 3 | 18 | 0.7895 | 0.5690 | 0.9419 | 934.13 | -1832.25 | -1738.66 | 0.00064 | 0.0970 | 0.1367 |
| **PG-V3** | **EQ-5D-5L VSE** | **PHQ-9, GAD-7** | 1339 | 4 | 25 | 0.7905 | 0.5336 | 0.9563 | **962.82** | **-1875.64** | -1745.65 | -0.00033 | 0.0963 | 0.1363 |
| PG-V4 | EQ-5D-5L VSE | PHQ-9 | 1339 | 2 | 10 | 0.7912 | 0.5483 | 0.9303 | 856.45 | -1692.91 | -1640.91 | -0.00108 | 0.0965 | 0.1370 |
| PG-V5 | EQ-5D-5L VSE | PHQ-9 | 1339 | 3 | 16 | 0.7904 | 0.5305 | 0.9319 | 926.03 | -1820.06 | -1736.86 | **-0.00025** | 0.0963 | 0.1365 |
| **PG-V6*** | **EQ-5D-5L VSE** | **PHQ-9** | 1339 | 4 | 22 | 0.7906 | 0.4836 | 0.9421 | 944.48 | -1844.96 | -1730.57 | -0.00043 | **0.0959** | **0.1361** |
| PG-V7 | EQ-5D-5L VSE | GAD-7 | 1339 | 2 | 10 | 0.7908 | 0.5443 | 0.9300 | 861.73 | -1703.47 | -1651.47 | -0.00067 | 0.0967 | 0.1368 |
| PG-V8 | EQ-5D-5L VSE | GAD-7 | 1339 | 3 | 16 | 0.7907 | 0.5426 | 0.9360 | 921.92 | -1811.84 | -1728.64 | -0.00056 | 0.0964 | 0.1364 |
| PG-V9 | EQ-5D-5L VSE | GAD-7 | 1339 | 4 | 22 | 0.7908 | 0.5314 | 0.9553 | 955.43 | -1866.85 | **-1752.46** | -0.00062 | 0.0965 | 0.1364 |
|  |  |  |  |  |  |  |  |  |  |  |  |  |  |  |
| PG-C1 | EQ-5D-5L Cross-walk | PHQ-9, GAD-7 | 1339 | 2 | 11 | 0.7228 | 0.4167 | 0.8874 | 609.27 | -1196.53 | -1139.33 | -0.00176 | 0.1235 | 0.1677 |
| PG-C2 | EQ-5D-5L Cross-walk | PHQ-9, GAD-7 | 1339 | 3 | 18 | 0.7209 | 0.4261 | 0.8877 | 739.80 | -1443.60 | -1350.01 | 0.00014 | 0.1240 | 0.1678 |
| **PG-C3** | **EQ-5D-5L Cross-walk** | **PHQ-9, GAD-7** | 1339 | 4 | 25 | 0.7219 | 0.3858 | 0.9115 | **811.19** | **-1572.37** | **-1442.38** | -0.00081 | 0.1233 | 0.1672 |
| PG-C4 | EQ-5D-5L Cross-walk | PHQ-9 | 1339 | 2 | 10 | 0.7239 | 0.4086 | 0.8889 | 597.96 | -1175.92 | -1123.92 | -0.00282 | 0.1233 | 0.1679 |
| PG-C5 | EQ-5D-5L Cross-walk | PHQ-9 | 1339 | 3 | 16 | 0.7210 | 0.4077 | 0.8835 | 729.20 | -1426.41 | -1343.21 | **0.00007** | 0.1238 | 0.1676 |
| **PG-C6*** | **EQ-5D-5L Cross-walk** | **PHQ-9** | 1339 | 4 | 22 | 0.7227 | 0.3699 | 0.9089 | 790.77 | -1537.54 | -1423.15 | -0.00158 | **0.1228** | **0.1671** |
| PG-C7 | EQ-5D-5L Cross-walk | GAD-7 | 1339 | 2 | 10 | 0.7233 | 0.4127 | 0.8874 | 603.22 | -1186.44 | -1134.45 | -0.00225 | 0.1235 | 0.1677 |
| PG-C8 | EQ-5D-5L Cross-walk | GAD-7 | 1339 | 3 | 16 | 0.7218 | 0.4294 | 0.8894 | 733.37 | -1434.74 | -1351.55 | -0.00075 | 0.1240 | 0.1678 |
| PG-C9 | EQ-5D-5L Cross-walk | GAD-7 | 1339 | 4 | 22 | 0.7233 | 0.3910 | 0.9087 | 798.00 | -1552.00 | -1437.61 | -0.00222 | 0.1235 | 0.1675 |

**Footnote.** All models used the same number of observations (N = 1340 or 1339) and the same within component variables (Xvars): PHQ-9, GAD-7. The best performing model specification within each performance statistic (i.e. LL, AIC, BIC, AE, MAE, and RMSE) is highlighted using **bold** font; the model number (Model No) is also highlighted in **bold** font in this instance; the final chosen model is marked with *.

**Variable types.** PHQ-9 and GAD-7 were classed as continuous variables.

**Acronyms.** AE, absolute error; AIC, Akaike information criteria; BIC, Bayesian information criteria; DF, degrees of freedom; EQ-5D-5L, EQ-5D five-level version; GAD-7, generalised anxiety disorder-7; LL, log likelihood; MAE, mean absolute error; PHQ-9, patient health questionnatire-9; ReQoL-UI, recovering quality of life – utility index; RMSE, root mean square error; VSE, value set for England.

**
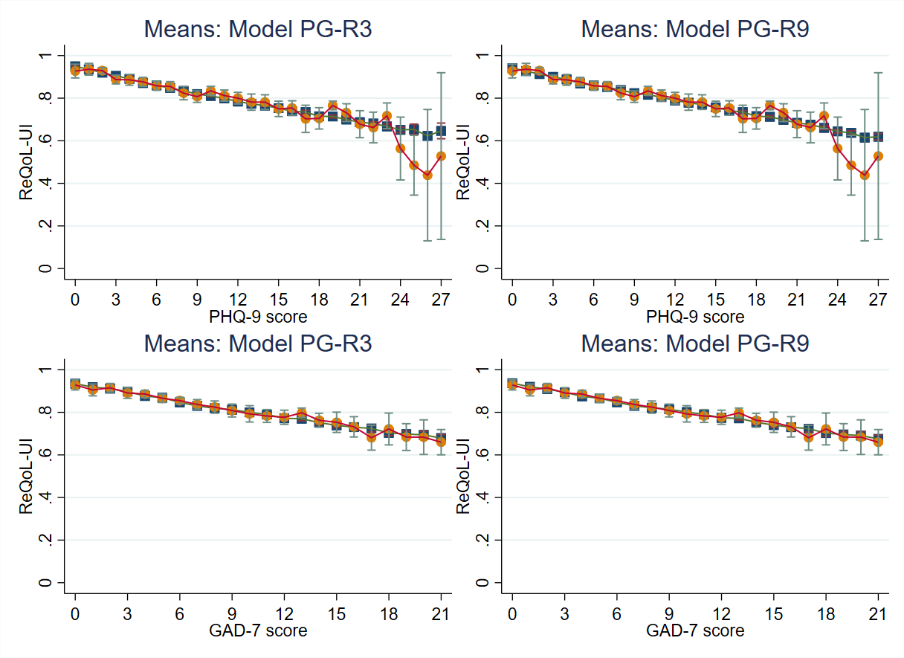
**

**
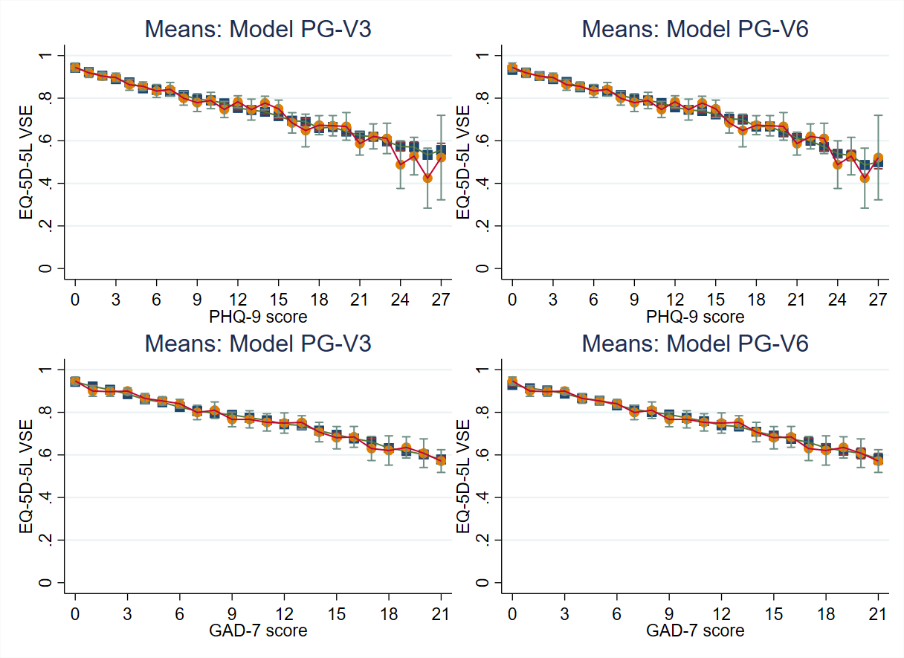
**

**
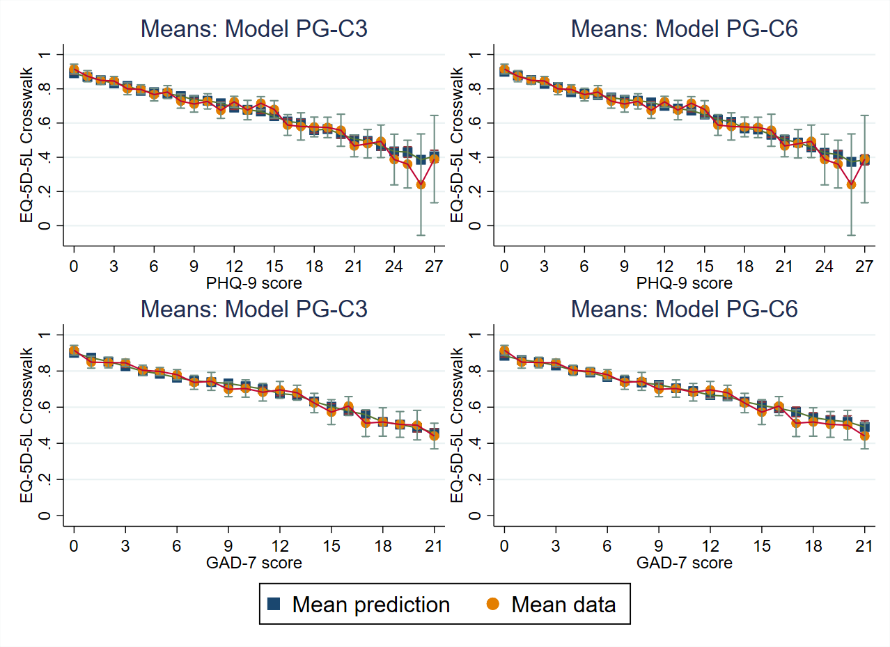
**

**Figure S2.5. Mean predicted and observed utility scores for PG-models: PG-R3 Vs PG-R9; PG-V3 Vs PG-V6; PG-C3 Vs PG-C6**

**
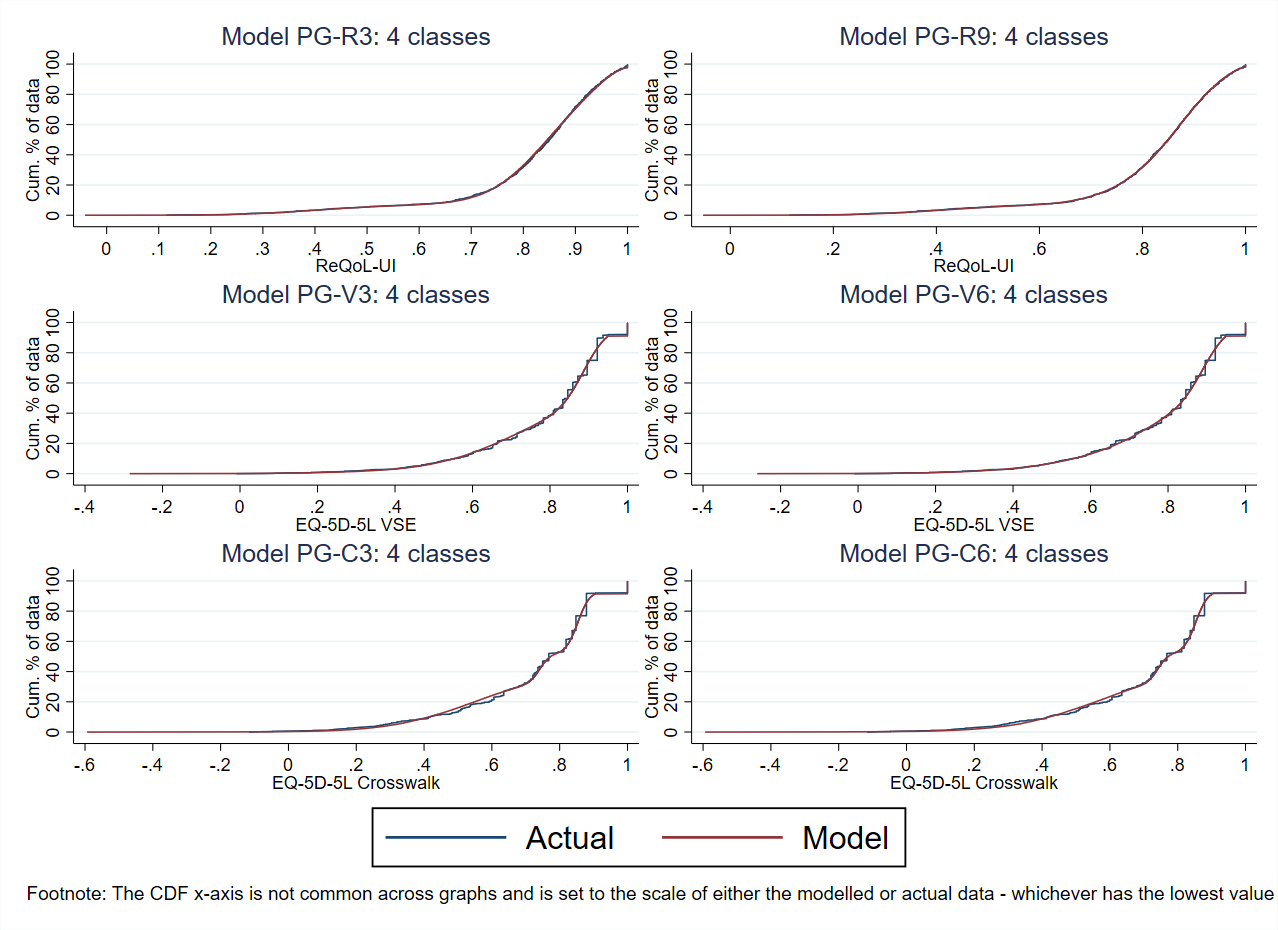
**

**Figure S2.6. Cumulative distribution functions for the simulated data for PG-models: PG-R3 Vs PG-R9; PG-V3 Vs PG-V6; PG-C3 Vs PG-C6**

**References**

1. Kroenke K, Spitzer RL, Williams JB, et al. Anxiety disorders in primary care: prevalence, impairment, comorbidity, and detection. Ann Intern Med. 2007; 146: 317-25.

2. Spitzer RL, Kroenke K, Williams JB, et al. A brief measure for assessing generalized anxiety disorder: the GAD-7. Arch Intern Med. 2006; 166: 1092-97.

3. Kroenke K, Spitzer RL, Williams JB. The PHQ‐9: validity of a brief depression severity measure. J Gen Intern Med. 2001; 16: 606-13.

4. Hernández Alava M, Wailoo A, Pudney S, et al. Mapping clinical outcomes to generic preference-based outcome measures: development and comparison of methods. Health Technology Assessment (Winchester, England). 2020; 24: 1.

5. Hernández Alava M, Wailoo A, Pudney S, et al. Modelling generic preference based outcome measures-development and comparison of methods. Health technology assessment. 2019.

6. Keetharuth A.D., Rowen D., Bjorner J., et al. Estimating a Preference-Based Index for mental health from the Recovering Quality of Life (ReQoL) measure: Valuation of ReQoL-UI. Value Health. 2020.

7. Keetharuth AD, Brazier J, Connell J, et al. Recovering Quality of Life (ReQoL): a new generic self-reported outcome measure for use with people experiencing mental health difficulties. Br J Psychiatry. 2018; 212: 42-49.

8. van Hout B, Janssen M, Feng Y-S, et al. Interim scoring for the EQ-5D-5L: mapping the EQ-5D-5L to EQ-5D-3L value sets. Value Health. 2012; 15: 708-15.

9. Devlin NJ, Shah KK, Feng Y, et al. Valuing health‐related quality of life: An EQ‐5D‐5L value set for England. Health Econ. 2018; 27: 7-22.

10. Pickard AS, Law EH, Jiang R, et al. United States valuation of EQ-5D-5L health states using an international protocol. Value in Health. 2019; 22: 931-41.

11. NHS Digital. A guide to IAPT data and publications. NHS Digital, 2021.
